# Curcumin reduces neuroinflammation and oxidative stress in a stroke model by epigenetically regulating *ADRB2* methylation through JAK2/STAT3 and Nrf2/HO-1 pathways

**DOI:** 10.1186/s12974-026-03729-y

**Published:** 2026-02-11

**Authors:** Liangzhe Wei, He Ren, Mingyue Zhao, Tianqi Xu, Jianhong Yang, Xinpeng Deng, Jie Sun, Shengjun Zhou, Jianmin Zhang, Xiang Gao, Yi Huang

**Affiliations:** 1 Ningbo Key Laboratory of Nervous System and Brain Function, Department of Neurosurgery, The First Affiliated Hospital of Ningbo University, Ningbo, Zhejiang 315010 China; 2 Department of Neurosurgery, The Second Affiliated Hospital, School of Medicine, Zhejiang University, Hangzhou, Zhejiang 310003 China; 3 South West Sydney Clinical Campuses, Faculty of Medicine & Health, University of New South Wales, Sydney, NSW 2170 Australia; 4 Ningbo Key Laboratory of Nervous System and Brain Function, Cerebrovascular Disease Center, The First Affiliated Hospital of Ningbo University, Ningbo, Zhejiang 315010 China; 5 Department of Neurosurgery, Beijing Hospital, National Center of Gerontology, Institute of Geriatric Medicine, Chinese Academy of Medical Sciences & Peking Union Medical College, Dongcheng, Beijing, 100730 China

**Keywords:** Acute ischemic stroke, Biomarkers, DNA methylation, *ADRB2*, Neuroinflammation, Oxidative stress, Epigenetics

## Abstract

The pathological mechanism of acute ischemic stroke (AIS) is complex, and exploring new diagnostic biomarkers and key molecular pathological targets is crucial for improving patient prognosis. The role of epigenetic regulation, especially DNA methylation, in AIS is receiving increasing attention, but its key target genes and clinical translational value still need to be elucidated. This study included 90 case-control groups and used pyrophosphate sequencing to detect the methylation level of β2-adrenergic receptor (*ADRB2*) promoter in peripheral blood. Using oxygen glucose deprivation/reperfusion (OGD/R) cell models and middle cerebral artery occlusion (MCAO) mouse models, the effects of *ADRB2* expression on JAK2/STAT3 and Nrf2/HO-1 pathways, as well as inflammatory and oxidative stress factors, were evaluated using Western blot, ELISA, and other techniques. Intervention study using curcumin. The *ADRB2* promoter region in the peripheral blood of AIS patients showed significant hypermethylation, and its level was significantly negatively correlated with *ADRB2* mRNA and protein expression. ROC curve analysis shows that the methylation level of specific CpG sites has extremely high predictive value for AIS diagnosis (AUC ≥ 0.9). Functional experiments have confirmed that DNMT1 mediated methylation of *ADRB2* increases after ischemia and hypoxia, and the downregulation of ADRB2 protein directly leads to activation of the JAK2/STAT3 pathway and inhibition of the Nrf2/HO-1 pathway, thereby synergistically exacerbating cerebral ischemic injury. Curcumin reverses the high methylation of *ADRB2* by inhibiting DNMT1, upregulates *ADRB2* expression, effectively improves neurological deficits, and reduces infarct volume, verifying the therapeutic feasibility of targeting this molecular pathological target. This study systematically elucidates for the first time that high methylation of *ADRB2* promoter is a new biomarker and key pathological mechanism node of AIS. The first discovery of curcumin exerting neuroprotective effects through epigenetic regulation of *ADRB2* expression provides a new strategy for early diagnosis and targeted therapy of AIS, as well as future targeted drugs targeting *ADRB2* methylation.

**Supplementary Information:**

The online version contains supplementary material available at 10.1186/s12974-026-03729-y.

## Introduction

Acute ischemic stroke (AIS) poses a major global health challenge, with high morbidity and mortality rates, ranking as the fifth leading cause of death worldwide [1]. Although vascular recanalization serves as the cornerstone of AIS treatment, reperfusion itself may induce ischemia-reperfusion (I/R) injury, thereby limiting clinical benefits [2]. Consequently, exploring new therapeutic targets and strategies to mitigate I/R damage has become crucial. Extensive research consistently demonstrates that neuroinflammation plays a pivotal role in the development and progression of ischemic brain injury [3–5]. Cerebral ischemia-reperfusion activates resident microglia and promotes peripheral immune cells to infiltrate into the infarct area and penumbra area. Proinflammatory mediators such as tumor necrosis factor (TNF), interleukin-1β (IL-1β) and interleukin-6 (IL-6) are up-regulated in the infarct area, and infiltrating cells and resident cells coordinate the inflammatory response after stroke [6]. At the same time, oxidative stress, as an independent injury factor, promotes lipid peroxidation and aggravates inflammatory response through excessive accumulation of reactive oxygen species (ROS) [7].

DNA methylation, an important epigenetic modification, has become an important participant in the occurrence and progression of ischemic stroke [8]. This mechanism usually involves the addition of methyl on the cytosine residue, which often occurs in the CpG island of the gene promoter region, which usually leads to transcriptional inhibition and affects protein function [9]. DNA methylation patterns are influenced by environmental factors. In humans, blood lipids and smoking can cause varying degrees of methylation changes [10]. Previous case-control studies have identified differentially methylated genes, such as *CDH2* and *PCDHB10*, as potential biomarkers for the diagnosis of ischemic stroke in the Chinese population [11]. Additionally, research has emphasized the important role of DNA methylation in large-artery atherosclerotic stroke, with *MTRNR2L8* methylation proposed as a potential therapeutic target and diagnostic biomarker [12]. Studies on *CDKN2B* DNA methylation levels in peripheral blood leukocytes of ischemic stroke patients suggest its possible involvement in arterial calcification [13].

β2-adrenergic receptor (*ADRB2*) is an important component of sympathetic nerve signal transmission in mammals, and is closely related to inflammation, oxidative stress, and even angiogenesis. Zahalka et al. proved that the absence of *ADRB2* in endothelial cells enhances oxidative phosphorylation, thereby inhibiting angiogenesis [14]. This finding emphasizes the important role of *ADRB2* in angiogenesis. In addition, Agac et al. Revealed that norepinephrine plays a role through β2-adrenergic receptor, inhibits the secretion of a variety of pro-inflammatory cytokines, and promotes the release of interleukin-10 (IL-10) through toll like receptor (TLR) signal transduction, significantly inhibiting the response of macrophages to lipopolysaccharide (LPS) [15]. In addition, studies have shown that blocking *ADRB2* in tumor cells can reduce the level of oxidative stress and stabilize the inflammatory response [16]. These convincing findings clearly prove the close relationship between *ADRB2* signaling and inflammation and oxidative stress. *ADRB2* has also been reported in ischemic stroke (IS), but its underlying mechanism is still poorly understood. A hospital-based case-control study by Kumar et al. showed that the Gln27Glu polymorphism of *ADRB2* gene may increase the risk of macrovascular disease stroke in northern India [17]. Similarly, Zhang et al. determined ADRB2 as a potential risk factor of is through bioinformatics analysis [18]. However, whether its expression is regulated by epigenetics in AIS, especially the direct regulation of DNA methylation in the promoter region, is currently unknown. Elucidating the methylation status and functional impact of *ADRB2* in AIS is expected to provide a new perspective for understanding stroke pathophysiology and potentially discover new diagnostic biomarkers.

Curcumin is a multi-target, multi-functional natural polyphenol compound, which is extracted from the rhizome of Curcuma longa. As the main active ingredient of turmeric, it shows a variety of pharmacological effects, including antioxidant, anti-inflammatory, anti-cancer and cardiovascular protective properties [19]. Recent studies have shown that curcumin can restore the expression and function of DNA methyltransferases (DNMTs), indicating that it is involved in epigenetic regulation [20]. However, its specific mechanism of action, especially whether it exerts its protective effect on AIS by intervening in epigenetic processes, is currently not well studied.

Therefore, the main purpose of this project is to study whether there are differences in the methylation level of *ADRB2* gene in AIS patients and whether the downstream ADRB2 protein expression changes. In addition, we will explore how these changes affect inflammation and oxidative stress, and clarify the potential molecular mechanisms. In addition, we will study whether curcumin can play anti-inflammatory and antioxidant roles in ischemic stroke through epigenetic regulation. This study aims to reveal potential therapeutic effects in regulating *ADRB2* mediated pathways and reducing stroke related injuries.

## Methods

### The author declares that all supporting data can be found in the article and its supplementary methods

#### Recruitment of clinical trial participants

The AIS patients in this study were all hospitalized in the Neurology Department of the First Affiliated Hospital of Ningbo University from January 2024 to August 2024, totaling 90 cases. The control group consisted of 90 hospitalized patients who were excluded from acute cerebrovascular disease, including hemifacial spasm, trigeminal neuralgia, Parkinson’s disease, etc. All patients signed informed consent forms and obtained approval from the Ethics Review Committee of the First Affiliated Hospital of Ningbo University. The inclusion and exclusion criteria for patients are detailed in the supplementary methods.

#### Patient clinical information, blood sample collection and biochemical analysis

Clinical information of the subjects was collected through detailed medical history interviews. It includes demographic data (gender, age, body mass index [BMI]), admission blood pressure, hypertension and diabetes history, smoking and drinking status, as well as related biochemical indicators (blood lipids, blood glucose levels) and hematological parameters (white blood cell count, etc.). All patients were taken to the hospital by ambulance upon onset of illness, and two 5mL blood samples were collected from all patients within 2 h of admission. A 5mL tube is used for immediate biochemical analysis. Measure biochemical indicators in the hospital biochemistry laboratory using a fully automated biochemical analyzer (Olympus AU2700, Tokyo, Japan). For specific biochemical indicators and blood processing procedures, please refer to the supplementary methods.

#### Phosphoric acid sequencing and cytokine detection

For quantitative DNA methylation analysis, the Qiagen Genomic DNA Extraction Kit (Cat. No. BJD4068, Hilden, Germany) was used to extract genomic DNA from peripheral blood leukocytes. The extracted DNA is then subjected to bisulfite conversion. Use PyroMark Assay Design software to design specific primer sequences for *ADRB2* pyrophosphate sequencing, and provide them in Supplementary Table 2. Measure the levels of cytokines (IL-2, IL-4, IL-6, TNF -α, IFN -γ, IL-10, and IL-17) in the plasma of AIS patients and control subjects using flow Cytometric Bead Array (CBA) method. According to the manufacturer’s instructions, perform CBA using the human Th1/Th2/Th17 cytokine assay kit (flow cytometry Luminex detection) (Cat. No. P010701001, Saiji Biotechnology, Nanchang, China). The specific methods for selecting methylation sites and CBA are detailed in the supplementary methods.

#### Cell culture, OGD/R model establishment, and cell drug treatment

Human brain microvascular endothelial cells (HBMECs) were purchased from Zhongqiao Xinzhou Biotechnology (Cat. No. ZQ0961, Shanghai, China), and an in vitro oxygen glucose deprivation/reperfusion (OGD/R) cell model was established. Throughout the experiment, control cells were cultured in complete medium under normoxic conditions. Based on previous literature, 5 µM 5-AZA (Cat. No. HY-A0004, MedChemExpress, New Jersey, USA) was selected for subsequent in vitro studies [21]. For curcumin treatment, based on subsequent cell viability assays and its ability to inhibit DNMT1, 7.5µM curcumin (Cat. No. HY-N0005, MedChemExpress, New Jersey, USA) was selected. In addition, according to the literature, 100nM salbutamol (Cat. No. HY-B1037, MedChemExpress, New Jersey, USA) [22] and 25µM ICI118551 (Cat. No. B1004, APExBIO, Houston, Texas, USA) were used [23]. After OGD/R, the cells were co incubated with 5-AZA, curcumin, salbutamol, and ICI118551 for 24 h before further analysis. Throughout the experiment, salbutamol was used as an agonist of ADRB2 protein, ICI 118551 as a highly selective antagonist of ADRB2, and 5-AZA as a recognized demethylating agent to compare whether curcumin exerts demethylation effects. The specific methods of cell culture and OGD/R, as well as the specific grouping of cells, are detailed in the supplementary methods.

#### Animal handling and ethics

Male C57BL/6 mice, weighing 24–26 g, purchased from Zhejiang Weitonglihua Laboratory Animal Technology. All animal care and experimental procedures strictly follow the guidelines established by the Institutional Animal Care and Use Committee of Ningbo University, which also provided ethical approval for this study. To minimize gender related variables, only male C57BL/6 mice were used. Animals are raised under controlled environmental conditions (22–24 °C, 40–60% humidity), with a 12-hour light/dark cycle and free access to food and water.

#### Establishment of MCAO model and drug treatment

Establish a middle cerebral artery occlusion (MCAO) model in mice. Anesthetize mice with 1.25% tribromoethanol via intraperitoneal injection, followed by surgery. According to the established protocol, administration was started immediately 1 h after reperfusion and continued for 3 consecutive days via intraperitoneal injection: 5-AZA group: 0.12 mg/kg [21], curcumin group: 150 mg/kg [24], salbutamol group: 15 mg/kg [22], ICI118551 group: 1 mg/kg [25]. According to the literature, the dosage of ML385 (Cat. No. B8300, APExBIO, Houston, Texas, USA) is 30 mg/kg [26], and the dosage of Coumermycin A1(Cat. No. HY-N7452, MedChemExpress, New Jersey, USA) is 100 µg/kg [27]. Administration was given every 24 h. Drug treatment should be performed within 72 h after treatment (acute phase), and brain tissue and peripheral blood should be obtained. The specific methods for establishing the MCAO model and drug grouping are detailed in the supplementary methods.

#### Cerebral blood perfusion imaging

Monitor cerebral blood flow perfusion in mice using a laser speckle contrast imaging system (Simopto, Wuhan, China). Collect blood perfusion images at three key time points in each MCAO mouse: immediately after insertion of the suture, immediately after withdrawal of the suture, and on the third day after MCAO. Mice with a reduction of ≥ 30% in cerebral blood flow (CBF) after inserting the suture compared to baseline were considered successful MCAO models and included in subsequent experiments [28]. The parameter settings and specific operation methods of the blood flow perfusion imaging instrument are detailed in the supplementary methods.

#### Measurement of infarct volume

During the acute phase of cerebral infarction (day 3), the infarct volume was evaluated by staining with 2,3,5-triphenyltetrazolium chloride (TTC) (Cat. No. HY-B1102, Solarbio, Beijing, China). Please refer to the supplementary methods for specific operation instructions. Quantitative analysis of infarct volume using ImageJ software.

#### Assessment of blood-brain barrier permeability

On the third day after MCAO, Evans blue (EB) (Cat. No. HY-B1102, MedChemExpress, New Jersey, USA) extravasation was used to assess blood-brain barrier (BBB) permeability. Collect brain tissue, slice and extract EB dye from the tissue, and quantify its concentration by measuring absorbance at 620 nm and comparing it with a standard curve of known EB concentration. Please refer to the supplementary methods for specific operation instructions.

#### Assessment of neurological deficits

Evaluate the neurological function of mice comprehensively using the Modified Neurological Severity Score (mNSS), Angle Test, Rotary Rod Test, and Adhesive Paper Removal Test. All tests were conducted on the day before MCAO, and then on the 1st, 3rd, 5th, and 7th day after MCAO. In all experiments, each animal was independently evaluated three times by two researchers who were unaware of the experimental conditions. The specific evaluation methods for each project are detailed in the supplementary methods.

#### Staining of live/dead cells and testing of cell viability

To evaluate the cell viability after OGD/R, Calcein AM/propidium iodide (PI) cell viability and cytotoxicity assay kit (Cat. No. C2015M, Beyotime Biotechnology, Shanghai, China) was used. After following the instructions provided by the reagent manufacturer, evaluate cell viability and cytotoxicity under a fluorescence microscope. Perform cell counting analysis using ImageJ software. To evaluate the effects of different concentrations of 5-AZA or curcumin on cell viability, a Cell Counting Kit-8 (CCK-8) assay was performed (Cat. No. RM02823, ABclonal, Wuhan, China). Measure the absorbance at 450 nm. The specific methods for staining live/dead cells and testing cell viability are detailed in the supplementary methods.

#### Cell transfection

In order to establish a small interfering RNA (siRNA) knockdown system for *ADRB2*, both targeted siRNA and non-targeted empty siRNA (control) were synthesized by Jima Company. Transfection was performed using Lipofectamine 3000 (Lip3000) (Cat. No. CN2541162, Thermo Fisher Scientific, MA, USA) as the transfection vector. Dilute siRNA (final concentration of 50nM) and Lip3000 in Opti MEM medium. Cells were transfected in Opti MEM medium for 6 h, and then switched to complete medium for further cultivation. In order to establish an *ADRB2* plasmid overexpression system, the *ADRB2* expression vector was synthesized by Jima Corporation. Dilute plasmid DNA (concentration of 500 ng/µL) with p3000 and Lipofectamine 3000 in Opti MEM medium. The subsequent transfection process is the same as the siRNA transfection process.

#### Enzyme linked immunosorbent assay (ELISA)

Process the collected cell culture supernatant and animal brain tissue homogenate according to the manufacturer’s instructions. Subsequently, the expression levels of IL-6, IL-10, IL-1β, and TNF -α were measured using species-specific ELISA kits. Measure the absorbance at 450 nm. Please refer to the supplementary methods for specific types of reagent kits.

#### Western blotting

After removing proteins from cells and animals, sample the same amount of proteins onto SDS-PAGE gel for electrophoresis. Then transfer the separated proteins onto a PVDF membrane. The membrane was sealed with 5% skim milk at room temperature and then incubated with corresponding primary and secondary antibodies. Visualize protein bands using chemiluminescence imaging and perform semi quantitative analysis using ImageJ software. For detailed operation steps and antibody information, please refer to the supplementary methods.

#### Real time quantitative polymerase chain reaction (RT-qPCR)

Extract total RNA from the sample using the RNA Rapid Extraction Kit (Cat. No. RN001, ESscience Biotech, Shanghai, China). Then, the RNA was reverse transcribed into cDNA using ABScribe III RT Master Mix (Cat. No. RK20429, ABclonal, Wuhan, China). Finally, use PerfectStart ^®^ Green qPCR SuperMix (Cat. No.AQ601, TransGen Biotech, Beijing, China) was subjected to RT qPCR on the LightCycler 480 system from Roche (Mannheim, Germany). The PCR primer sequences are provided in Supplementary Table 2. Use the 2-^ΔΔ^ CT method to calculate the relative expression level between the given sample and the reference sample.

#### Immunofluorescence staining

Cell and tissue samples were fixed with 4% paraformaldehyde (Cat. No. P1110, Solarbio, Beijing, China) and permeabilized with 0.5% Triton X-100 (Cat. No. T8200, Solarbio, Beijing, China). Nonspecific binding was blocked at room temperature using immunofluorescence blocking solution (Cat. No. P0206, Beyotime Biotechnology, Shanghai, China). Subsequently, the corresponding primary and secondary antibodies were incubated and stained with DAPI (Cat. No. S2110, Solarbio, Beijing, China). Fluorescence images were obtained using a fluorescence microscope and analyzed using ImageJ software. For detailed operation steps and antibody information, please refer to the supplementary methods.

#### Detection of oxidative stress markers

Measure the levels of lipid peroxidation (malondialdehyde, MDA), reduced glutathione (GSH), and superoxide dismutase (SOD) activity using commercially available test kits according to the manufacturer’s instructions. Quantify these markers using a Microplate Reader. Using CheKine ™ The reactive oxygen species (ROS) content detection kit (fluorescence method) (Cat. No. KTB1911, Abbkine, Wuhan, China) was used to detect the ROS content in cells. The fluorescence signal intensity of DCF in live cells was observed using a fluorescence microscope, and image analysis was performed using ImageJ. The specific methods and kit information for testing are detailed in the supplementary methods.

#### Statistical analysis

All data were analyzed using GraphPad Prism statistical analysis software (Version 8.3.0, CA, USA). Two sets of normally distributed data were compared using the two tailed unpaired Student’s t-test, while non normally distributed data were tested using the Mann Whitney U rank sum test. Use one-way ANOVA for component comparison, and post hoc Dunnett’s test or Tukey’s test to correct for P-values. Perform correlation analysis using Pearson correlation. Use receiver operating characteristic (ROC) curves to analyze the predictive value of methylation for cerebral infarction. All data were measured as mean ± standard error (SEM), with *P* < 0.05 indicating statistically significant differences.

## Results

### Clinical characteristics and *ADRB2* DNA methylation levels in AIS patients

**Fig. 1:**
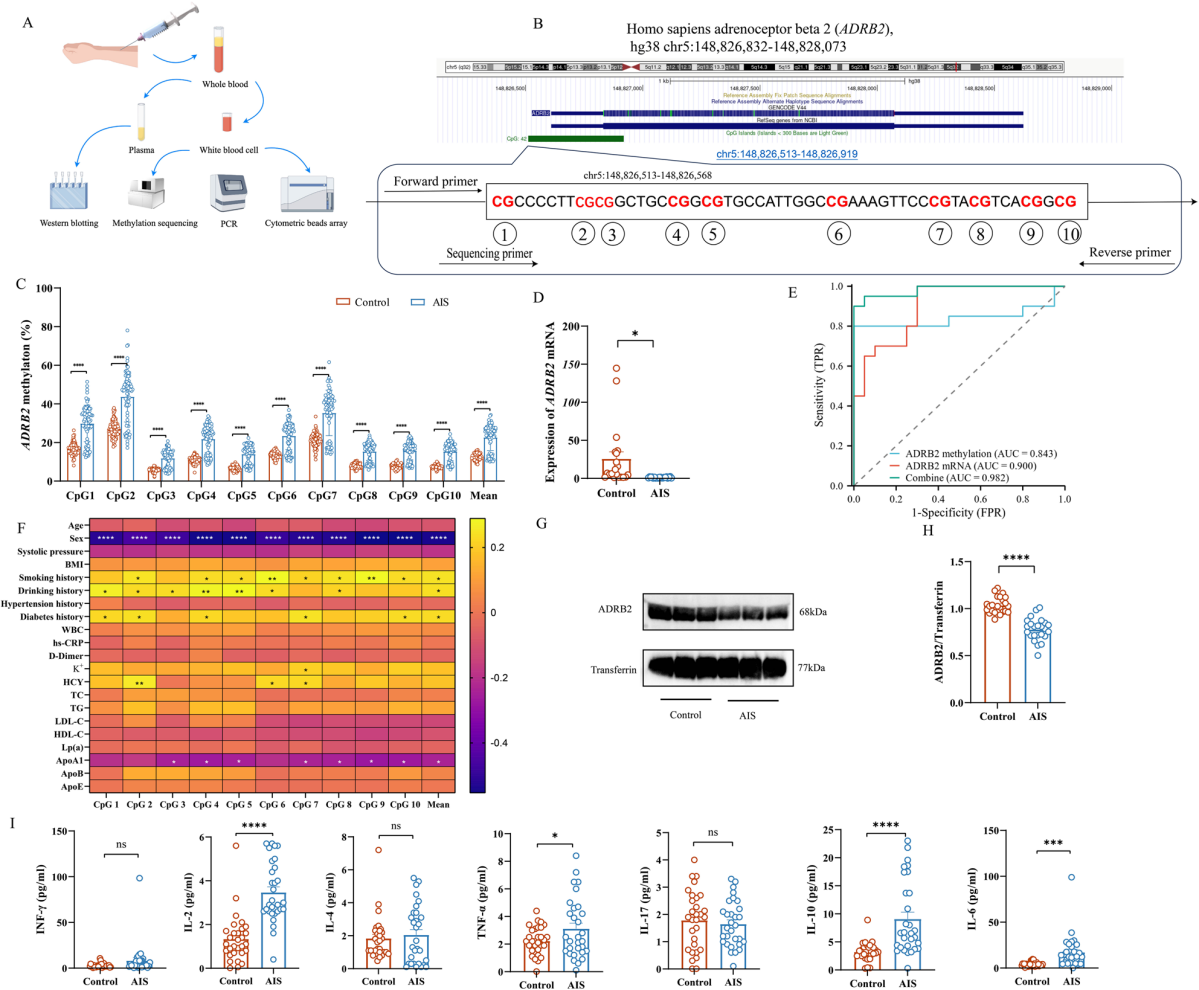
Increased *ADRB2* gene DNA methylation, decreased *ADRB2* mRNA and protein expression, and elevated plasma inflammatory cytokines following AIS. **A** Flowchart of the clinical study. **B** Distribution of the target sequences and 10 CpG sites located in the promoter region of *ADRB2*. **C** Detection of *ADRB2* DNA methylation at CpG sites 1–10 using pyrosequencing on white blood cells collected from participants. *n* = 90. **D** RT-qPCR analysis of *ADRB2* mRNA levels in white blood cells from participants. *n* = 24. **E** Receiver operating characteristic (ROC) curves for average methylation of *ADRB2* CpG sites, *ADRB2* mRNA, and combined mRNA and *ADRB2* methylation. **F** Heatmap of correlation analysis between 21 clinical variables and biochemical indicators with methylation levels. *n* = 90. **G** Representative Western blot bands and density quantification of ADRB2 protein in plasma. The relative levels of each protein were normalized to transferrin from the same sample for Western blot analysis. **H** Density quantification of ADRB2 protein bands. *n* = 24. **I **Quantification of cytokines (IFN-γ, IL-2, IL-4, TNF-α, IL-17, IL-10, IL-6) in plasma using the Cytometric Bead Array method. *n* = 30. Data in (**C**) were analyzed using two-way ANOVA, while other data were analyzed using the Mann-Whitney test. All data are expressed as means ± SEM. ns indicates no significant difference, *P < 0.05, **P < 0.01, ***P < 0.001, ****P < 0.0001. In Fig. 1F, the white * represents negative correlation, and the black * represents positive correlation

This study enrolled 90 patients with AIS (45 males, 45 females, mean age 65.81 ± 0.86 years) and 90 age and gender matched controls (45 males, 45 females, mean age 64.27 ± 0.84 years, *p* > 0.05) (Table 1). Significant statistical differences were observed between AIS and control groups for systolic blood pressure, total cholesterol (TC), low-density lipoprotein (LDL), high-density lipoprotein (HDL), lipoprotein (a) (Lp(a)), apolipoprotein A1 (ApoA1), and apolipoprotein B (ApoB). The AIS group also presented with a higher proportion of patients with a history of smoking, diabetes, and hypertension. While BMI, homocysteine (HCY), triglyceride (TG), and apolipoprotein E (ApoE) levels showed no significant differences between groups (Table 1), upon admission, AIS patients exhibited higher systolic blood pressure, HCY, and Lp(a) levels compared to controls. On the contrary, the levels of LDL cholesterol, HDL cholesterol, ApoA1, ApoB and ApoE in AIS group were lower. In order to study the epigenetic modification, a segment of *ADRB2* gene (chr5:148826513–148826568) was selected for methylation sequencing. Pyromark assay design software identified 10 CpG sites in this region for quantitative methylation analysis (Fig. 1B). Statistical analysis showed that compared with the control group, the methylation levels of all 10 single CpG sites and the average methylation levels in AIS patients were significantly higher (*P* < 0.0001) (Fig. 1C). Hierarchical analysis by gender confirmed that the methylation level of *ADRB2* gene was increased in both male and female AIS patients (Fig. S1A-S1B).

**Table 1:** Comparison of clinical characteristics between the cerebral infarction group and the control group

Characteristics	AIS (*n* = 90)	Control (*n* = 90)	t/χ2	*P*
Age (year)	65.81 ± 0.86	64.27 ± 0.84	1.284	0.201
BMI (kg/㎡)	24.37 ± 0.35	23.47 ± 0.48	1.435	0.154
Systolic pressure (mmHg)	142.3 ± 2.15	130.9 ± 2.82	3.182	**0.002**
Sex (female), n	45	45	/	/
Smoking, n	17	9	/	/
Drinking, n	16	16	/	/
Hypertension, n	70	18	/	/
Diabetes, n	67	6	/	/
HCY (µmol/L)	12 ± 0.57	9.73 ± 0.48	1.886	0.061
TC (mmol/L)	4.28 ± 0.11	4.86 ± 0.09	3.885	**0.0001**
TG (mmol/L)	1.45 ± 0.09	1.61 ± 0.10	1.506	0.139
LDL-C (mmol/L)	2.80 ± 0.08	3.04 ± 0.08	2.563	**0.011**
HDL-C (mmol/L)	1.08 ± 0.02	1.27 ± 0.03	2.424	**0.016**
Lp(a) (mg/L)	175.9 ± 18.81	27.26 ± 7.36	6.589	**<0.0001**
ApoA1 (g/L)	1.17 ± 0.02	1.35 ± 0.03	2.521	**0.013**
ApoB (g/L)	0.74 ± 0.02	0.92 ± 0.03	2.112	**0.036**
ApoE (mg/L)	45.60 ± 2.14	47.79 ± 1.95	0.751	0.454

*WBC* White blood cell, *HCY* Homocysteine, *TC* Total cholesterol, *TG* Triglycerides, *LDL-C* Low-density lipoprotein cholesterol, *HDL-C* High-density lipoprotein cholesterol, *Lp(a)* Lipoprotein(a), *ApoA1* Apolipoprotein A1, *ApoB* Apolipoprotein B, *ApoE* Apolipoprotein E. Continuous data are expressed as mean ± standard error (SEM). Ordinal data are presented as count (*n*). Bold values indicate *P* < 0.05 (statistically significant)

In addition, RT-qPCR analysis showed that compared with the control group, *ADRB2* mRNA expression in AIS patients was significantly decreased (Fig. 1D), suggesting that there may be a negative correlation between *ADRB2* methylation and its expression in AIS. ROC curve analysis was performed to evaluate the predictive value of *ADRB2* methylation (CpG sites 1–10) and mRNA expression for the diagnosis of AIS. The area under the curve (AUC) of CpG7 was 0.7777, indicating that it had medium predictive value. The AUC values of CpG1, CpG2, CpG3, CpG4, CpG6, CpG8 and the average methylation level were between 0.8 and 0.9, indicating that it has high predictive value for AIS. It is worth noting that the AUC values of CpG5, CpG9 and CpG10 are ≥ 0.9, indicating an excellent predictive value for AIS (Fig. S1C). This finding is confirmed by the strong predictive value of *ADRB2* mRNA expression (AUC = 0.900) (Fig. 1E). In addition, the AUC value of average methylation was 0.843, while the AUC value of the combination of *ADRB2* mRNA expression and average *ADRB2* methylation level was 0.982 (Fig. 1E), indicating that their combined use has a strong predictive value for AIS.

### Correlation between *ADRB2* gene methylation levels and clinical data

Given that environmental factors can influence the development and progression of AIS by regulating gene expression through DNA methylation in promoter regions, we evaluated the association between *ADRB2* gene methylation and 23 high-risk clinical factors. These factors included sex, smoking history, drinking history, hyperlipidemia, hypertension, diabetes, BMI, and HCY levels (Fig. 1F). Significant differences in *ADRB2* gene methylation were observed with respect to sex, smoking history, drinking history, diabetes history, serum potassium levels, HCY and ApoA1 levels (*P* < 0.05) (Fig. S2A-M). Specifically, smoking history, drinking history, diabetes history, serum potassium level and HCY were positively correlated with *ADRB2* gene methylation level. In contrast, ApoA1 levels were inversely correlated with *ADRB2* methylation. In addition, male AIS patients showed higher *ADRB2* gene methylation levels than female patients. No significant correlation was found between other factors and methylation levels at any CpG site.

### Reduced ADRB2 protein levels and elevated inflammatory markers in AIS patient blood

To further elucidate the changes in ADRB2 levels in the blood of patients, Western blot analysis of plasma protein concentrations was performed. The results showed that ADRB2 protein levels were significantly reduced in AIS patients compared with controls (Fig. 1G-H). In parallel, we assessed inflammatory cytokine levels in the patients’ plasma using a flow-cytometry-based bead array. AIS patients exhibited significantly elevated levels of IL-2, IL-6, TNF-α, and IL-10 compared to controls. No significant differences were observed in IL-4, IL-17, and IFN-γ levels (Fig. 1I). These findings demonstrate a correlation between elevated inflammatory cytokines and decreased ADRB2 protein expression in patients with acute ischemic stroke.

### *ADRB2* downregulation in the OGD/R model

**Fig. 2:**
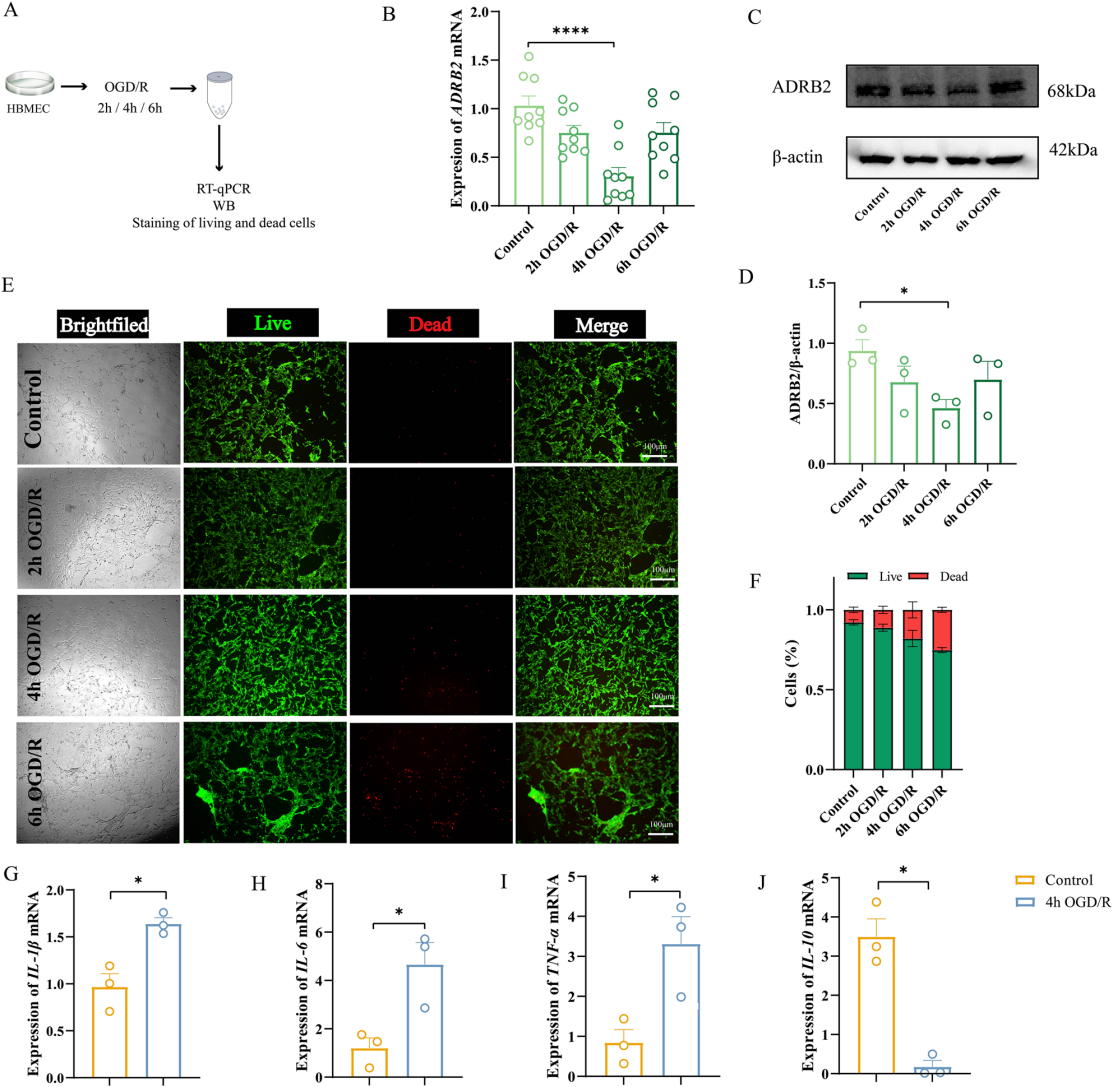
Determination of OGD/R time points for cell studies. **A** Flowchart. **B** RT-qPCR analysis of *ADRB2* mRNA expression in HBMECs after 2 h, 4 h, and 6 h OGD/R. *n* = 9. **C** Representative Western blot bands for ADRB2 in HBMECs cell lysates after 2 h, 4 h, and 6 h OGD/R. The relative protein levels were normalized to β-actin from the same sample for Western blot analysis. **D** Density quantification of ADRB2 bands. *n* = 3. **E** Representative images of live/dead cell staining in HBMECs after 2 h, 4 h, and 6 h OGD/R. Scale bar, 100 μm. **F** Quantification of cell numbers from live/dead staining images. *n* = 3. **G**-**J** RT-qPCR analysis of *IL-1β* (**G**), *IL-6* (**H**), *TNF-α* (**I**), *IL-10 *(**J**) mRNA expression in HBMECs after OGD/R. *n* = 3. Data in (**B**) and (**D**) were analyzed using one-way ANOVA, and post hoc Dunnett’s test to correct for *P*-values., while other data were analyzed using Student’s t-test. All data are expressed as means ± SEM. *P < 0.05, **P < 0.01, ***P < 0.001, ****P < 0.0001

To elucidate the role of *ADRB2* in ischemic injury, we employed an in vitro OGD/R model using HBMECs to simulate cerebral ischemia-reperfusion injury. RT-qPCR measurements of *ADRB2* mRNA expression were performed at various time points post-OGD/R. Results showed a downward trend in *ADRB2* mRNA expression following OGD/R, with statistically significant reduction observed at 4 h compared to the control group (Fig. 2B). Western blot analysis further confirmed that ADRB2 protein expression decreased at different time points during OGD/R, reaching its lowest point at 4 h before showing an upward trend (Fig. 2C-D). These findings suggest that *ADRB2* expression is dynamically regulated after ischemic injury and may play a role in regulating ischemia-reperfusion injury and subsequent repair processes.

Live/dead cell staining showed that the overall cell viability decreased at different OGD/R time points within 6 h. However, viable cells remained dominant (Fig. 2E-F). This confirmed that our OGD/R treatment did not induce significant cell death in HBMECs, allowing the cellular response to be studied. Therefore, the 4 h OGD/R time point was chosen for subsequent *ADRB2* expression studies as it represents the most significant reduction in *ADRB2* expression. At this 4 h time point, RT-qPCR results showed increased mRNA levels of proinflammatory cytokines (*IL-1β*, *IL-6*, *TNF-α*) (Fig. 2G-I) and decreased mRNA expression of the anti-inflammatory cytokine *IL-10* (Fig. 2J). This suggests that the significant reduction in *ADRB2* expression coincides with the peak inflammatory response at this specific time point after OGD/R.

*ADRB2* is closely related to an anti-inflammatory phenotype in the OGD/R model

**Fig. 3:**
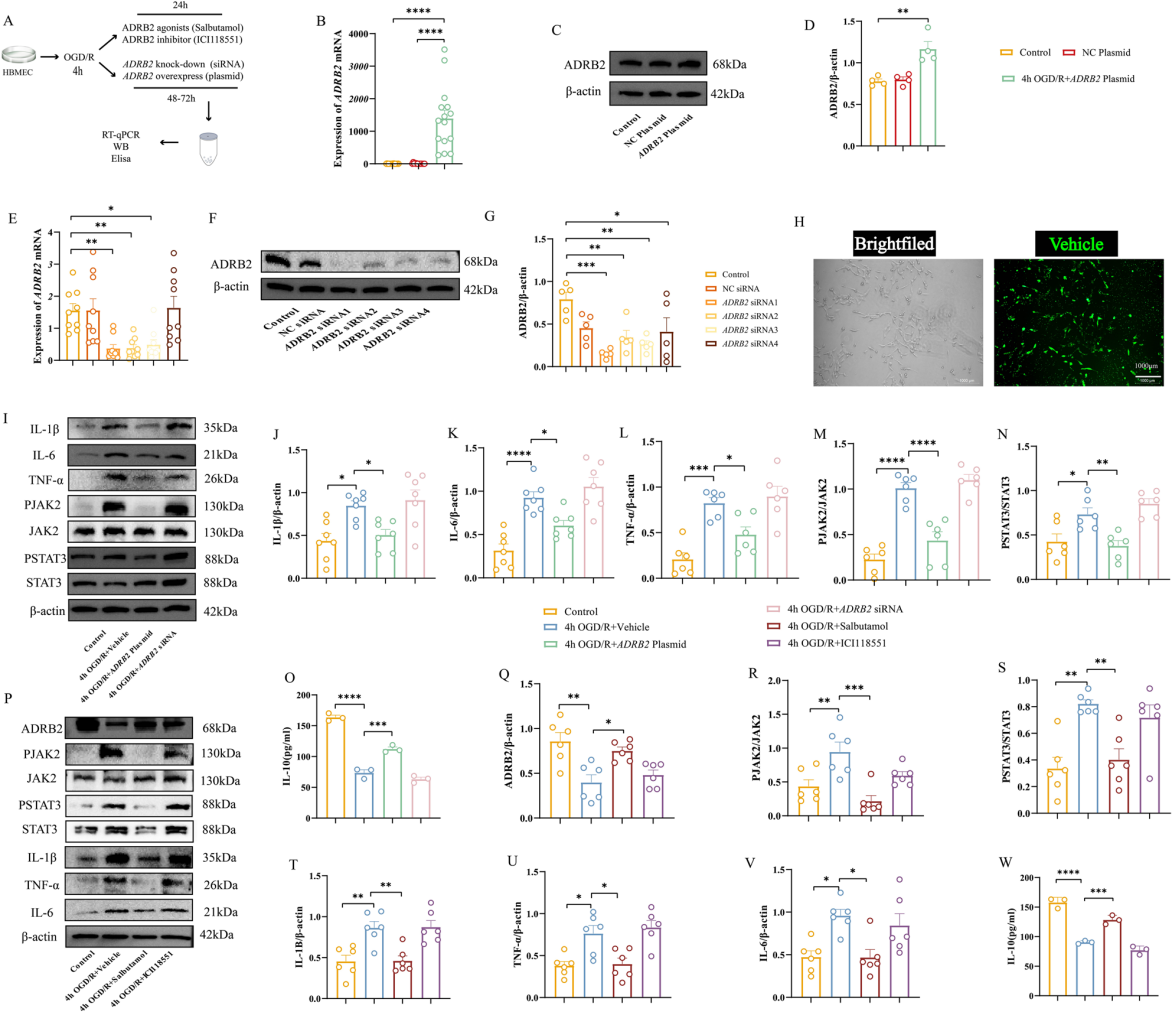
*ADRB2* gene is closely associated with inflammation in OGD/R-induced HBMECs. **A** Flowchart. **B** RT-qPCR validation of successful plasmid overexpression of *ADRB2*. *n* = 15. **C** Representative Western blot bands for ADRB2 in HBMECs cell lysates following *ADRB2* plasmid overexpression. The relative protein levels were normalized to β-actin from the same sample for Western blot analysis. **D** Density quantification of ADRB2 bands. *n* = 4. **E** RT-qPCR validation of successful *ADRB2* knockdown using siRNA. *n* = 9. **F** Representative Western blot bands for ADRB2 in HBMECs cell lysates following *ADRB2* knockdown with siRNA. The relative protein levels were normalized to β-actin from the same sample for Western blot analysis. **G** Density quantification of ADRB2 bands. *n* = 5. **H** Representative images of siRNA and plasmid transfection labeled with fluorescent vector. Green indicates successful transfection of siRNA and plasmid into cells. Scale bar, 1000 μm. **I** Representative Western blot bands for p-JAK2, JAK2, p-STAT3, STAT3, IL-1β, IL-6, and TNF-α in cell lysates from OGD/R-induced HBMECs treated with empty vector, *ADRB2* knockdown siRNA, and *ADRB2* overexpression plasmid. The relative protein levels were normalized to β-actin from the same sample for Western blot analysis. **J**-**N** Density quantification of IL-1β (**J**), IL-6 (**K**), TNF-α (**L**), p-JAK2 and JAK2 (**M**), p-STAT3 and STAT3. **N**. *n* = 6. **O** ELISA analysis of inflammatory cytokines IL-10 in the supernatants of OGD/R-induced HBMECs treated with empty vector, *ADRB2* knockdown siRNA, and *ADRB2* overexpression plasmid. *n* = 3. **P** Representative Western blot bands for ADRB2, p-JAK2, JAK2, p-STAT3, STAT3, IL-1β, TNF-α, and IL-6 in cell lysates from OGD/R-induced HBMECs treated with solvent DMSO, ADRB2 agonist Salbutamol, and ADRB2 antagonist ICI118551. The relative protein levels were normalized to β-actin from the same sample for Western blot analysis. **Q**-**V** Density quantification of ADRB2 (**Q**), p-JAK2 and JAK2 (**R**), p-STAT3 and STAT3 (**S**), IL-1β (**T**), TNF-α (**U**), IL-6 **V**. *n* = 6. **W** ELISA analysis of inflammatory cytokines IL-10 in the supernatants of OGD/R-induced HBMECs treated with solvent DMSO, ADRB2 agonist Salbutamol, and ADRB2 antagonist ICI118551. *n* = 3. Data were analyzed using one-way ANOVA, and post hoc Dunnett’s test to correct for *P*-values. All data are expressed as means ± SEM. *P < 0.05, **P < 0.01, ***P < 0.001, ****P < 0.0001

We next investigated the correlation between inflammatory factor changes and *ADRB2* expression following OGD/R by constructing *ADRB2* siRNA knockdown and overexpression models. Transfection efficiency was confirmed by RT-qPCR, Western blot, and fluorescence vector analysis. *ADRB2* overexpression significantly elevated both its mRNA and protein expression levels (Fig. 3B-D). For knockdown, four different siRNA sequences were tested; siRNA1, siRNA2, siRNA3 and siRNA4 significantly reduced *ADRB2* mRNA levels by RT-qPCR (Fig. 3E), with siRNA1 showing the most pronounced decrease in protein expression (Fig. 3F-G). Fluorescence vector analysis confirmed successful cellular transfection (Fig. 3H).

Western blot analysis showed that the levels of pro-inflammatory cytokines (IL-1β, IL-6, TNF-α) were significantly higher in the OGD/R group than in the control group. Compared with the OGD/R group, *ADRB2* overexpression significantly reduced the expression of pro-inflammatory factors IL-1β, IL-6, and TNF-α. Although no statistically significant differences were observed in the *ADRB2* knockout group, pro-inflammatory factors showed a marked upward trend compared to the OGD/R group (Fig. 3I and J-L). Meanwhile, *ADRB2* overexpression significantly increased the anti-inflammatory factor IL-10 (Fig. 3O). These results demonstrate that while OGD/R-induced *ADRB2* downregulation coincides with elevated inflammatory factor expression, *ADRB2* overexpression enhances the anti-inflammatory activity of HBMECs.

### JAK2/STAT3 axis is involved in *ADRB2*-mediated anti-neuroinflammatory response after OGD/R

To further elucidate the mechanism by which *ADRB2* regulates inflammatory factors, we investigated the JAK2/STAT3 signaling pathway. Western blot analysis revealed that the OGD/R group exhibited increased JAK2 phosphorylation (p-JAK2), which subsequently led to elevated STAT3 phosphorylation (p-STAT3) compared to the control group. Overexpression of *ADRB2* attenuated these OGD/R-induced increases in p-JAK2 and p-STAT3 expression. In contrast, *ADRB2* knockdown resulted in an upward trend in p-JAK2 and p-STAT3 expression (Fig. 3I and M-N).

To determine whether *ADRB2* acts upstream of the JAK2/STAT3 axis or has a direct/indirect relationship with this pathway, we treated cells exposed to OGD/R with an ADRB2 agonist (salbutamol) and a highly selective ADRB2 antagonist (ICI118551). Salbutamol treatment significantly inhibited the p-JAK2 and p-STAT3 (Fig. 3P and R-S). In addition, the expression levels of the downstream proinflammatory cytokines IL-1β, IL-6, and TNF-α were significantly decreased after salbutamol treatment (Fig. 3P and T-V). In addition, the anti-inflammatory factor IL-10 was significantly increased after salbutamol administration (Fig. 3W). These data strongly suggest that the JAK2/STAT3 axis is a key mediator of *ADRB2*-mediated anti-neuroinflammatory activity.

### *ADRB2* participates in the antioxidant response after OGD/R via the Nrf2/HO-1 pathway

**Fig. 4:**
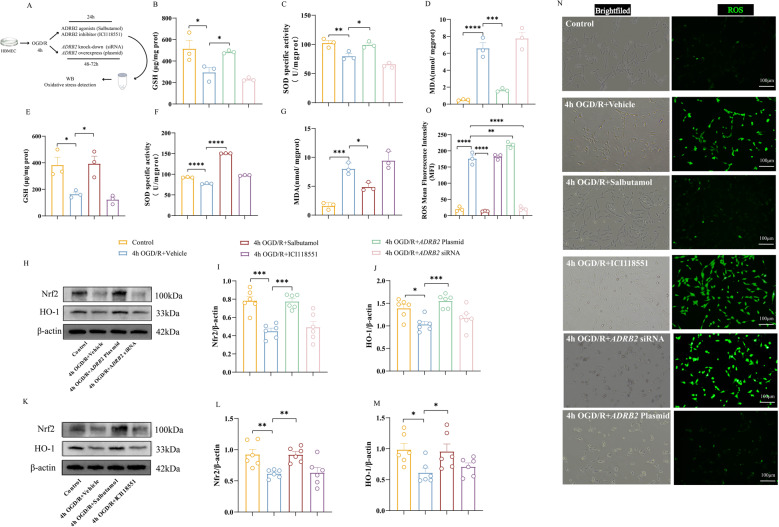
*ADRB2* gene is closely associated with oxidative stress in OGD/R-induced HBMECs. **A** Flowchart. **B**-**D** Expression levels of oxidative damage markers GSH (**B**), SOD (**C**), and MDA (**D**) in OGD/R-induced HBMECs treated with empty vector, *ADRB2* knockdown siRNA, and *ADRB2* overexpression plasmid. *n* = 3. **E**-**G** Expression levels of oxidative damage markers GSH (**E**), SOD (**F**), and MDA (**G**) in OGD/R-induced HBMECs treated with solvent DMSO, ADRB2 agonist Salbutamol, and ADRB2 antagonist ICI118551. *n* = 3. **H** Representative Western blot bands for Nrf2 and HO-1 in cell lysates from OGD/R-induced HBMECs treated with empty vector, *ADRB2* knockdown siRNA, and *ADRB2* overexpression plasmid. The relative protein levels were normalized to β-actin from the same sample for Western blot analysis. **I**-**J** Density quantification of Nrf2 (**I**) and HO-1 (**J**) proteins. *n* = 6. **K** Representative Western blot bands for Nrf2 and HO-1 in cell lysates from OGD/R-induced HBMECs treated with solvent DMSO, ADRB2 agonist Salbutamol, and ADRB2 antagonist ICI118551. The relative protein levels were normalized to β-actin from the same sample for Western blot analysis. **L**-**M** Density quantification of Nrf2 (**L**) and HO-1 (**M**) proteins. *n* = 6. **N** Representative fluorescence images of ROS in OGD/R-induced HBMECs treated with solvent DMSO, ADRB2 agonist Salbutamol, ADRB2 antagonist ICI118551, *ADRB2* knockdown siRNA, and *ADRB2* overexpression plasmid. White represents the bright-field image, and green indicates the fluorescence intensity of ROS. Scale bar, 100 μm. **O** Quantification of fluorescence ROS intensity. *n* = 3. Data were analyzed using one-way ANOVA, and post hoc Dunnett’s test to correct for *P*-values. All data are expressed as means ± SEM. *P < 0.05, **P < 0.01, ***P < 0.001, ****P < 0.0001

To verify the relationship between *ADRB2* activation and antioxidant stress, we assessed the levels of Nrf2, the antioxidant enzyme HO-1, and the intracellular content of reduced GSH, MDA, ROS, and SOD. After OGD/R treatment, MDA and ROS levels increased, while SOD and GSH levels decreased compared with the control group. After overexpression of *ADRB2*, GSH and SOD levels were significantly increased (Fig. 4B-C), while MDA levels were decreased (Fig. 4D), and ROS levels were also decreased (Fig. 4N-O). Western blot results further confirmed that *ADRB2* overexpression significantly upregulated Nrf2/HO-1 expression (Fig. 4H-J). Further, treatment with salbutamol in cells exposed to OGD/R resulted in increased GSH and SOD expression (Fig. 4E-F), decreased MDA levels (Fig. 4G), and attenuated ROS fluorescence intensity (Fig. 4N-O). Western blot analysis confirmed that Nrf2/HO-1 expression was down-regulated under OGD/R compared to control, and the difference was statistically significant. Salbutamol treatment upregulated Nrf2/HO-1 expression, whereas inhibition of ADRB2 with ICI118551 resulted in a decrease in Nrf2/HO-1 levels (Fig. 4K-M). These findings together confirm the existence of a direct or indirect regulatory relationship between *ADRB2* and the Nrf2/HO-1 pathway.

Therefore, our results suggest that OGD/R-induced decrease in *ADRB2* expression is accompanied by an increase in oxidative stress markers and a decrease in antioxidant enzyme activity, and that enhancing *ADRB2* expression is effective in attenuating oxidative stress damage in HBMECs.

### Optimization of 5-AZA and curcumin concentrations and their effects on epigenetic modifiers and ADRB2 in OGD/R model

**Fig. 5:**
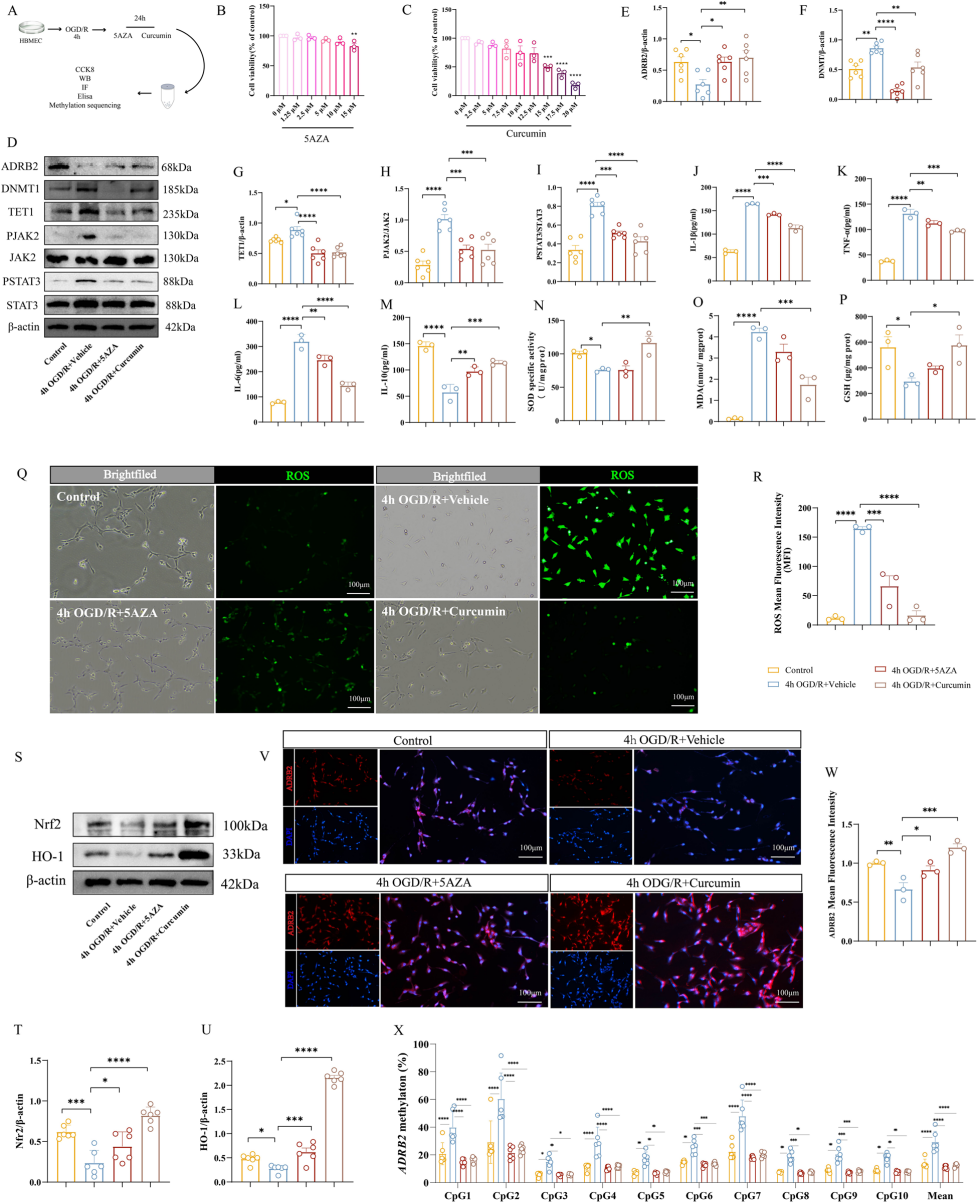
Curcumin and 5-AZA treatment ameliorate inflammation and oxidative stress in OGD/R-induced HBMECs. **A** Flowchart. **B** CCK-8 assay of HBMECs viability treated with different concentrations of 5-AZA. *n* = 3. **C** CCK-8 assay of HBMECs viability treated with different concentrations of curcumin. *n* = 3. **D** Representative Western blot bands for ADRB2, DNMT1, TET1, p-JAK2, JAK2, p-STAT3, and STAT3 in cell lysates of OGD/R-induced HBMECs treated with DMSO, 5-AZA, or curcumin. The relative levels of each protein were normalized to β-actin from the same sample for Western blot analysis. **E**-**I** Quantification of ADRB2(E), DNMT1 (**F**) TET1(**G**), p-JAK2 and JAK2(**H**), p-STAT3 and STAT3 **I**. *n* = 6. **J**-**M** ELISA analysis of inflammatory cytokines IL-1β (**J**), TNF-α (**K**), IL-6 (**L**) and IL-10 (**M**)in the supernatants of OGD/R-induced HBMECs treated with DMSO, 5-AZA, or curcumin. *n* = 3. **N**-**P** Expression levels of oxidative stress markers SOD (**N**), MDA (**O**), and GSH (**P**) in OGD/R-induced HBMECs treated with DMSO, 5-AZA, or curcumin. *n* = 3. **Q** Representative fluorescent ROS images of OGD/R-induced HBMECs treated with DMSO, 5-AZA, or curcumin, with white indicating bright field images and green representing the fluorescence intensity of ROS. Scale bar, 100 μm (**R**) Quantification of fluorescent ROS intensity. *n* = 3. **S** Representative Western blot bands for Nrf2 and HO-1 in cell lysates of OGD/R-induced HBMECs treated with DMSO, 5-AZA, or curcumin. The relative levels of each protein were normalized to β-actin from the same sample for Western blot analysis. **T**-**U** Quantification of Nrf2 (**T**) and HO-1 (**U**) protein density. *n* = 6. **V** Representative immunofluorescence images for ADRB2 protein (red fluorescence) in OGD/R-induced HBMECs treated with DMSO, 5-AZA, or curcumin. Scale bar, 100 μm (**W**) Quantification of the average fluorescence intensity of ADRB2. *n* = 3. **X** Pyrosequencing analysis of methylation levels at the *ADRB2* CpG1-10 sites in OGD/R-induced HBMECs treated with DMSO, 5-AZA, or curcumin. *n* = 6. Data are presented as means ± SEM, with statistical analysis performed using one-way ANOVA, and post hoc Dunnett’s test to correct for *P*-values. *P < 0.05, **P < 0.01, ***P < 0.001, ****P < 0.0001

5-AZA is a well-known drug that can reduce DNA methylation. The appropriate concentration of 5-AZA in HBMECs has not been fully determined. We chose 1.25µM, 2.5µM, 5µM, 10µ M, and 15µM of 5-AZA to treat OGD/R induced human microvascular endothelial cells. The CCK-8 results showed that there was no significant effect on the activity of HBMECs at various concentrations of 5-AZA, with IC50 values above 50% (Fig. 5B). We chose 5µM of 5-AZA for subsequent experiments.

DNMT1 is an enzyme that promotes DNA methylation, and studies have shown that 5-AZA is a specific inhibitor of DNA methyltransferase. WB results showed a significant increase in DNMT1 expression after OGD/R, and 5-AZA can inhibit the elevated state of DNMT1 after OGD/R (Fig. 5D and F). However, after intervention, TET1 protein showed a consistent trend with DNMT1 protein (Fig. 5D and G). Meanwhile, we found that 5-AZA can increase the expression level of ADRB2 protein after OGD/R (Fig. 5D and E).

We further observed the changes in DNMT1 and TET1 using different concentrations of 5-AZA. Compared with the OGD/R group, 5-AZA at various concentrations had a significant inhibitory effect on DNMT1, and the inhibitory effect increased with increasing concentration. TET1 expression increased at low concentrations and showed a decreasing trend with increasing concentration. When treated with 5µM 5-AZA, the difference was significant compared with the OGD/R group (Fig. S3A, S3C-D). Meanwhile, with the increase of 5-AZA, the content of ADRB2 gradually increased (Fig. S3A-B). Therefore, we chose 5-AZA as a comparative drug to investigate whether curcumin can affect methylation and the degree of methylation.

In our previous clinical experiments, we have discovered the phenomenon of high methylation of *ADRB2*, low expression of ADRB2 protein, and elevated plasma inflammatory factors after ischemic stroke. Through in vitro studies, we have elucidated the close relationship between *ADRB2* and inflammation and oxidative stress. Therefore, we speculate whether curcumin can affect epigenetic regulation, affect the expression of ADRB2 protein, and thus play a role in reducing neuroinflammation and oxidative stress. However, the appropriate concentration of curcumin in HBMECs has not been fully determined. To determine the optimal concentration of curcumin for HBMECs, we selected curcumin at concentrations of 2.5µM, 5µM, 7.5µM, 10µM, 12.5µM, 15µM, 17.5µM, and 20µM to treat OGD/R induced human brain microvascular endothelial cells. Curcumin had no significant effect on the cell viability of HBMECs between 2.5µM and 12.5µM, with IC50 values ranging from 12.5µM to 15µM. After exceeding 15µM, cell viability was significantly affected (Fig. 5C). We chose curcumin at 7.5µM for subsequent experiments.

We speculate that curcumin also has an inhibitory effect on DNMT1. The results showed that the inhibitory effect of curcumin was weaker than that of 5-AZA (Fig. 5D and F). At the same time, we found that curcumin can also increase the expression level of ADRB2 protein after OGD/R (Fig. 5D and E). Observe the changes of DNMT1 and TET1 with different concentrations of curcumin. Compared with the OGD/R group, with the increase of curcumin concentration, the inhibition of DNMT1 became more and more significant, and the difference in concentration above 7.5µM was statistically significant. The change in TET1 expression level by curcumin also showed significant differences at a concentration of 7.5µM (Fig. S3E, S3G-S3H). Meanwhile, with the increase of curcumin, the content of ADRB2 gradually increased (Fig. S3E-S3F). These data indicate that curcumin can inhibit DNMT1 protein, affect the expression level of TET1, and increase the content of ADRB2 protein.

#### Curcumin and 5-AZA treatment reduce OGD/R-Induced inflammation and oxidative stress in HBMECs via ADRB2

To evaluate the anti-inflammatory and antioxidant effects of curcumin and 5-AZA in vitro, we measured ROS, oxidative stress markers, and inflammatory cytokines in HBMECs. ELISA and ROS fluorescence results showed that OGD/R significantly elevated pro-inflammatory cytokines (IL-1β, IL-6, TNF-α) compared to the control group (Fig. 5I-L). Furthermore, the ROS fluorescence signal in the OGD/R group was markedly more intense, indicating increased ROS production in HBMECs following OGD (Fig. 5Q-R). Meanwhile, antioxidant factors such as GSH and SOD decreased, while oxidative damage markers like MDA increased (Fig. 5N-P).

Both 5-AZA and curcumin treatment effectively reversed the OGD/R-induced elevation of pro-inflammatory cytokines (Fig. 5I and L) and reduction of anti-inflammatory cytokine IL-10 (Fig. 5M), while significantly decreasing ROS fluorescence intensity, indicating reduced ROS production (Fig. 5Q and R). Specifically, curcumin treatment led to increased GSH and SOD levels and decreased MDA levels (Fig. 5N and P). Immunofluorescence staining further demonstrated that both curcumin and 5-AZA treatments resulted in higher ADRB2 fluorescence intensity compared to the OGD/R group, indicating increased ADRB2 protein expression (Fig. 5V and W).

Methylation sequencing revealed that treatment with curcumin and 5-AZA reduced methylation levels at CpG sites 1–10 of the *ADRB2* gene (Fig. 5X). In contrast, previously tested ADRB2 agonists (Salbutamol) and antagonists (ICI118551) showed no effect on *ADRB2* methylation (Fig. S4A), highlighting the epigenetic specificity of curcumin and 5-AZA.

These results indicate that curcumin and 5-AZA have potential anti-inflammatory and antioxidant stress effects in vitro, which are related to the reduced methylation status of *ADRB2* gene in OGD/R-induced HBMECs after treatment with 5-AZA and curcumin, thereby increasing the expression of ADRB2 protein.

### JAK2/STAT3 and Nrf2/HO-1 signal transduction mediate anti-inflammatory and antioxidant effects of curcumin and 5-AZA in HBMECs

To further explore the mechanism of phenotype changes. The WB results showed that treatment with 5-AZA and curcumin significantly reduced the high phosphorylation status of JAK2 and STAT3 after OGD/R (Fig. 5D and H-I), and the high phosphorylation status of JAK2 and STAT3 was the direct cause of downstream inflammatory cytokine accumulation. At the same time, we found that after treatment with 5-AZA and curcumin, the expression level of Nrf2/HO-1 significantly increased, especially in the curcumin group (Fig. 5S-U).

Based on previous experiments, we can conclude that treatment with 5-AZA and curcumin can reduce the methylation level of *ADRB2* gene after ischemia-reperfusion, and improve neurological function through ADRB2/p-JAK2/p-STAT3 and Nrf2/HO-1 axis.

### Grouping and mortality of MCAO mice

To further clarify the in vivo role of *ADRB2* in AIS and the efficacy of our interventions, we utilized the MCAO animal model. A total of 339 male C57BL/6 mice were used in this study (Supplementary Table 1). Among these, 294 mice underwent MCAO surgery, while 45 mice underwent sham surgery. The overall mortality rates were10.2% (30/294) for the MCAO group and 0.0% (0/45) for the sham group. A total of 51 MCAO mice were excluded from the study due to one of the following reasons: insufficient cerebral blood flow reduction in the ipsilateral hemisphere (less than 30% decrease compared to the contralateral side as measured by Laser Speckle Flow Imaging; *n* = 21), or death during or immediately after surgery (*n* = 30).

#### Curcumin and 5-AZA improve neurological function and reduce infarct volume after MCAO

**Fig. 6:**
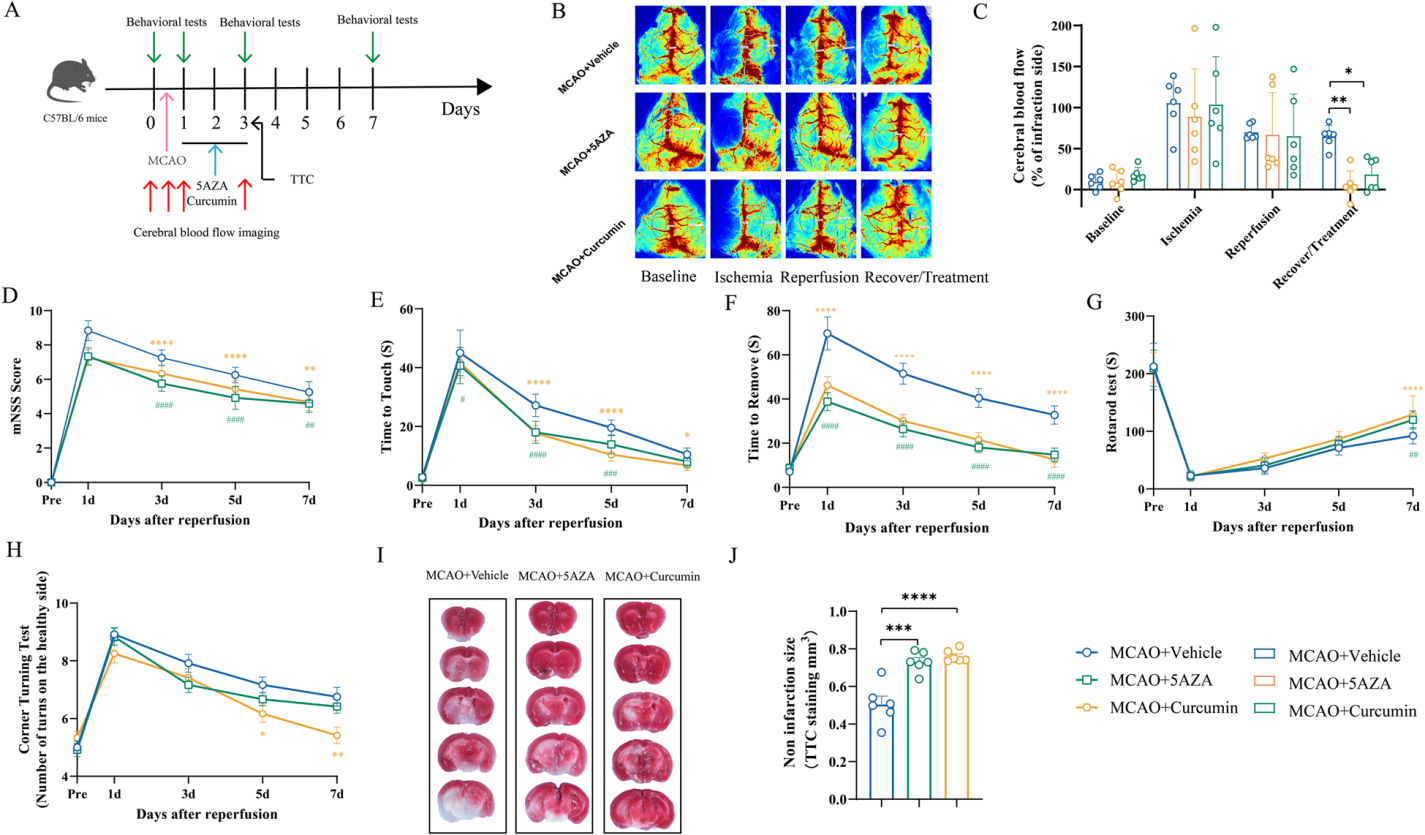
Curcumin and 5-AZA treatment promote recovery of neurological function and cerebral blood flow in MCAO mice. **A** Flowchart. **B**-**C**) Representative cerebral blood flow perfusion images (**B**) and quantification (**C**) obtained using laser speckle contrast imaging before MCAO, after MCAO filament insertion, after filament removal, and after treatment with DMSO, 5-AZA, or curcumin three days post-MCAO. The quantification calculates the ratio of reduction in blood flow on the damaged side compared to the normal side. *n* = 6. **D**-**H** Neurological function assessment in mice using mNSS score (**D**), time to touch (**E**), time to remove (**F**), rotarod test (**G**), and corner turning test **H**. Curcumin and 5-AZA treatment resulted in faster recovery of neurological function compared to the DMSO-treated MCAO group. *n* = 12. **I**-**J** Representative TTC staining images of coronal brain sections from MCAO mice treated with DMSO, curcumin, or 5-AZA (**I**) and semi-quantification of infarct areas **J**. *n* = 6. All data are presented as means ± SEM, with statistical analysis performed using one-way ANOVA, and post hoc Dunnett’s test to correct for *P*-values. MCAO+Vehicle vs. MCAO+Curcumin: *P < 0.05, **P < 0.01, ***P < 0.001, ****P < 0.0001; MCAO+Vehicle vs. MCAO+5AZA: #*P* < 0.05, ##*P* < 0.01, ###*P* < 0.001, ####*P* < 0.0001

We assessed the recovery of MCAO mice treated with 5-AZA and curcumin using comprehensive neurological scoring, 2,3,5-triphenyltetrazolium chloride (TTC) staining for infarct volume, and CBF perfusion imaging. The modified neurological severity score (mNSS), rotarod test, corner test, and adhesive removal test were performed prior to MCAO and on days 1, 3, 5, and 7 post-MCAO. Before MCAO, no significant differences in neurological function were observed between groups. However, all MCAO groups exhibited significant neurological deficits the day after MCAO. Importantly, between days 3 and 7 post-MCAO, both MCAO + 5-AZA and MCAO + Curcumin treatment groups demonstrated better neurological recovery than the MCAO + Vehicle control group. Notably, curcumin treatment caused less neurological impairment than 5-AZA (Fig. 6D-H).

TTC staining can evaluate the vitality of brain tissue and the degree of ischemic injury. The proportion of non-infarct volume in the MCAO group mice was 50.63%, and the non infarct volumes after treatment with 5-AZA and curcumin were 73.15% and 76.13%, respectively, showing significant differences compared to the MCAO + Vehicle group mice (Fig. 6I-J). In addition, laser speckle flow imaging showed a significant decrease in blood flow on the affected side compared to the healthy side after inserting a thread plug into the middle cerebral artery of mice. We selected MCAO mice with a decrease of more than 30% on the affected side compared to the healthy side as experimental mice. It is interesting that the MCAO + 5-AZA treatment group mice and MCAO + Curcumin treatment group mice showed better blood flow recovery and significantly improved vascular recanalization ability compared to the MCAO + Vehicle group mice. Although the MCAO + Vehicle group mice showed a trend of self-recovery of blood flow over time, there were significant differences compared to the treatment group mice (Fig. 6B-C). These data indicate that treatment with 5-AZA and curcumin can improve the results of neurological function tests, enhance blood flow during recovery after MCAO, and reduce infarct volume.

### Curcumin and 5-AZA reduce oxidative stress injury after Ischemia-Reperfusion in MCAO mice

**Fig. 7:**
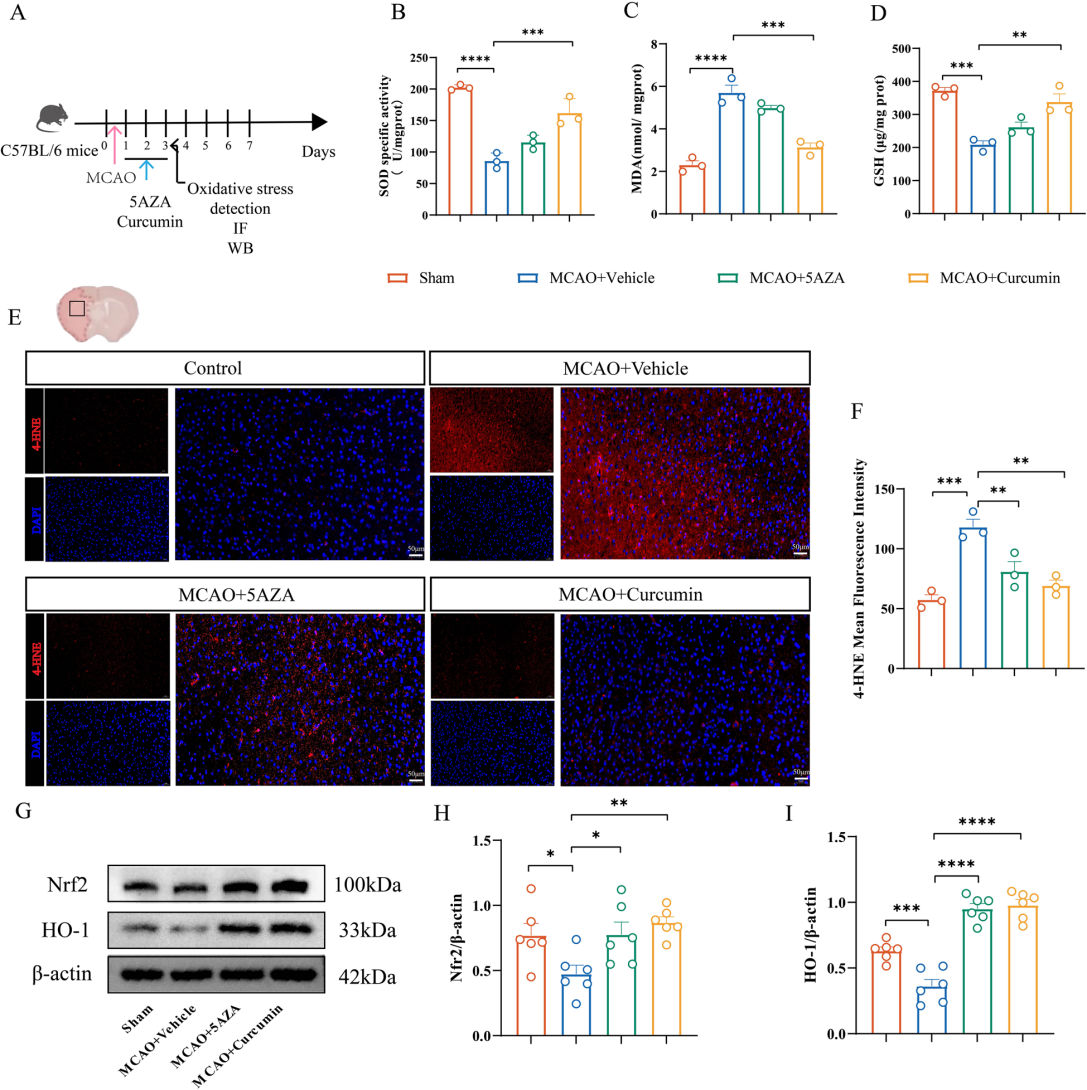
Curcumin and 5-AZA treatment alleviate oxidative stress damage in MCAO mice. **A** Flowchart. **B**-**D** Expression levels of oxidative stress markers SOD (**B**), MDA (**C**), and GSH (**D**) in the infarcted area of MCAO mice treated with DMSO, 5-AZA, or curcumin. *n* = 3. **E** Representative immunofluorescence images of the infarcted area in MCAO mice treated with DMSO, 5-AZA, or curcumin, with red indicating fluorescence intensity of 4-HNE protein. Scale bar, 50 μm (**F**) Quantification of the average fluorescence intensity of 4-HNE. *n* = 3. **G** Representative Western blot bands for Nrf2 and HO-1 in brain tissue lysates from the infarcted area of MCAO mice treated with DMSO, 5-AZA, or curcumin. The relative levels of each protein were normalized to β-actin from the same sample for Western blot analysis. **H**-**I** Quantification of Nrf2 (**H**) and HO-1 (**I**) protein density. *n* = 6. All data are presented as means ± SEM, with statistical analysis performed using one-way ANOVA, and post hoc Dunnett’s test to correct for *P*-values. *P < 0.05, **P < 0.01, ***P < 0.001, ****P < 0.0001

Oxidative stress is one of the important pathophysiological processes in cerebral ischemia-reperfusion injury. We measured the levels of MDA, GSH, and SOD in brain tissue to evaluate oxidative stress levels, as well as the expression of the oxidative stress pathway Nrf2/HO-1. The SOD and GSH levels in the MCAO + Curcumin group were significantly higher than those in the MCAO + Vehicle group, while the MDA levels were significantly lower than those in the treatment group. Compared with the sham group, the SOD and GSH content in MCAO mice decreased, and the MDA level increased, indicating a high level of oxidative stress in brain tissue. Curcumin treatment can reduce oxidative stress damage in MCAO mice (Fig. 7B-D).

Due to technical limitations in observing ROS in animal brain tissue, we chose lipid peroxidation product 4-HNE to indirectly evaluate the ROS content in brain tissue. The immunofluorescence results showed that the enhanced fluorescence intensity of 4-HNE in the MCAO + Vehicle group indicated increased oxidative stress, while the fluorescence intensity of 4-HNE decreased after treatment with 5-AZA and curcumin (Fig. 7E-F). At the same time, treatment with 5-AZA and curcumin can significantly increase the expression level of Nrf2/HO-1 (Fig. 7G-I), playing a protective role in oxidative stress injury. These data indicate that curcumin and 5-AZA treatment promote functional recovery by inhibiting oxidative stress damage.

### Curcumin and 5-AZA reduce neuroinflammation after ischemia-reperfusion in MCAO mice

**Fig. 8:**
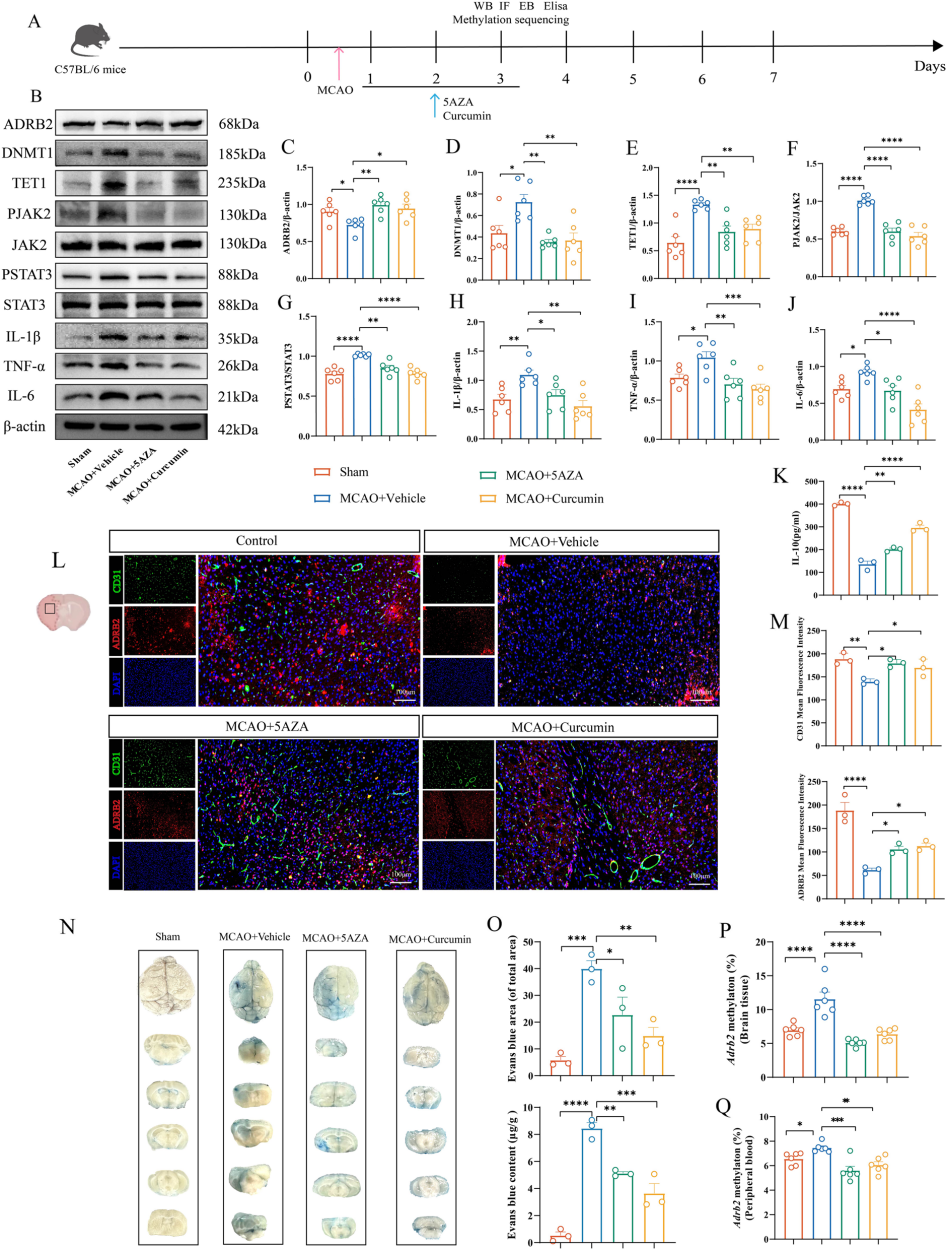
Curcumin and 5-AZA treatment reduce neuroinflammation, accelerate blood-brain barrier repair, and promote neovascularization in MCAO mice. **A** Flowchart. **B** Representative Western blot bands for ADRB2, DNMT1, TET1, p-JAK2, JAK2, p-STAT3, STAT3, IL-1β, IL-6, and TNF-α in brain tissue lysates from the infarcted area of MCAO mice treated with DMSO, 5-AZA, or curcumin. The relative levels of each protein were normalized to β-actin from the same sample for Western blot analysis. (C-J) Quantification of ADRB2 (**C**), DNMT1 (**D**), TET1 (**E**), p-JAK2 and JAK2 (**F**), p-STAT3 and STAT3 (**G**), IL-1β (H), TNF-α (**I**), IL-6 (**J**) protein densities. *n* = 6. **K** ELISA analysis of inflammatory cytokines IL-10 in brain tissue lysates from the infarcted area of MCAO mice treated with DMSO, 5-AZA, or curcumin. *n* = 3. **L** Representative immunofluorescence images of coronal brain sections from MCAO mice treated with DMSO, curcumin, or 5-AZA, stained with antibodies against ADRB2 (red), CD31 (green), and DAPI to assess angiogenesis. Images are from the infarct boundary, with scale bar = 100 μm. **M** Quantification of ADRB2 and CD31 immunofluorescence intensity. *n* = 3. **N** Representative images of blood-brain barrier disruption in coronal brain sections from MCAO mice treated with DMSO, curcumin, or 5-AZA. **O** Quantification of EB dye extravasation area and content in brain tissue. *n* = 3. **P** Pyrosequencing analysis of *Adrb2* methylation levels in the infarcted area brain tissue of MCAO mice treated with DMSO, curcumin, or 5-AZA. *n* = 6. **Q** Methylation sequencing results of *Adrb2* in peripheral blood of mice. All data are presented as means ± SEM, with statistical analysis performed using one-way ANOVA, and post hoc Dunnett’s test to correct for *P*-values. *P < 0.05, **P < 0.01, ***P < 0.001, ****P < 0.0001

Inflammatory responses are critical in cerebral ischemia-reperfusion injury. Therefore, we investigated changes in common pro-inflammatory cytokines (TNF-α, IL-6, IL-1β) in MCAO mice on postoperative day 3 and after three consecutive days of 5-AZA and curcumin treatment. Compared with the sham surgery group, the MCAO + Vehicle group showed significantly elevated levels of TNF-α, IL-6, and IL-1β (Fig. 8B, H and J). Crucially, both 5-AZA and curcumin treatment significantly reversed the elevated TNF-α, IL-6, and IL-1β expression in MCAO mice (Fig. 8B and H-J). Concurrently, anti-inflammatory cytokine IL-10 levels increased (Fig. 8K). Although curcumin consistently demonstrated stronger anti-inflammatory activity than 5-AZA, no statistically significant differences were observed between the two treatments. These data indicate that 5-AZA and curcumin effectively inhibit the release of inflammatory mediators during ischemia-reperfusion, thereby providing neuroprotective effects in MCAO mice.

We hypothesize that the upregulation of pro-inflammatory cytokines following MCAO in mice may be mediated by alterations in the JAK2/STAT3 signaling pathway. WB analysis showed that compared with the sham group, the MCAO + Vehicle group mice had increased expression of p-JAK2, further activating p-STAT3 and increasing accumulation of inflammatory factors. Compared with the MCAO + Vehicle group mice, the expression levels of p-JAK2 and p-STAT3 were downregulated after treatment with 5-AZA and curcumin (Fig. 8B and F-G). These studies suggest that 5-AZA and curcumin may inhibit p-JAK2 and p-STAT3 and exert anti-inflammatory activity.

### Curcumin and 5-AZA promote angiogenesis and accelerate BBB repair after MCAO

Cerebral ischemia-reperfusion is frequently accompanied by vascular endothelial injury, which subsequently triggers vascular endothelial inflammation. To evaluate vascular repair following curcumin and 5-AZA treatment, we performed immunofluorescence staining to detect the expression of ADRB2 and vascular endothelial marker CD31. Results showed that compared with the sham group, ADRB2 fluorescence intensity decreased and CD31 fluorescence intensity weakened after MCAO. However, both ADRB2 and CD31 fluorescence intensities increased to varying degrees following 5-AZA and curcumin treatment (Fig. 8L-M), suggesting that 5-AZA and curcumin may have potential to improve ischemia-reperfusion vascular endothelial injury, and indicating possible correlations between ADRB2 and CD31.

Endothelial cells are an important component of the blood-brain barrier, and the inflammatory factors released by endothelial cells during cerebral ischemia-reperfusion can affect the integrity of the blood-brain barrier. Electron beam (EB) staining revealed increased EB dye infiltration into brain tissue following MCAO, indicating barrier disruption. However, treatment with curcumin and 5-AZA significantly reduced EB dye leakage (Fig. 8N-O), demonstrating that these agents accelerate MCAO-induced blood-brain barrier repair.

### Curcumin and 5-AZA inhibit DNMT1 and increase ADRB2 protein expression in MCAO mice

In preliminary in vitro studies, we demonstrated that curcumin and 5-AZA modulate DNMT1 expression, thereby altering ADRB2 protein levels following OGD/R. This finding was further validated in animal models. In MCAO mice, ADRB2 expression was significantly reduced compared to the sham group. Conversely, treatment with 5-AZA and curcumin resulted in increased ADRB2 protein expression compared to the MCAO + Vehicle group (Fig. 8B-C).

Further investigation into its potential regulatory mechanisms revealed that MCAO-induced elevation of DNMT1 activity indicates hypermethylation of DNA, leading to hypermethylation and reduced protein expression in specific genes such as *Adrb2*. Notably, 5-AZA and curcumin significantly inhibit DNMT1 activity, exerting demethylation effects (Fig. 8B and D), thereby increasing ADRB2 protein expression.

TET1 is a key enzyme maintaining methylation status, which shows significant changes after MCAO, 5-AZA, and curcumin treatment. However, these TET1 alterations may simply reflect the dynamic balance between methylation and demethylation in vivo, rather than being direct targets of 5-AZA and curcumin therapy (Fig. 8B and E). Methylation sequencing of *Adrb2* CpG sites in mouse brain tissue confirmed that both curcumin and 5-AZA significantly reduced *Adrb2* methylation levels after MCAO (Fig. 8P). Meanwhile, we collected peripheral blood from MCAO mice via cardiac puncture. Results showed that 5-AZA and curcumin treatment significantly decreased *Adrb2* methylation in peripheral blood (Fig. 8Q), indicating consistency between peripheral blood and brain tissue methylation patterns. The target sequences and CpG sites of *Adrb2* in mice are illustrated in Fig. S4B.

These findings demonstrate that 5-AZA and curcumin enhance ADRB2 expression by inhibiting DNMT1 and reducing *Adrb2* gene methylation. This epigenetic regulation subsequently alleviates endothelial inflammation and oxidative stress damage in MCAO mice. This discovery not only corroborates in vitro cell experiments showing curcumin’s ability to suppress *Adrb2* methylation, but also sheds light on the phenomena observed in clinical samples.

### ADRB2 mediates anti-inflammatory and antioxidant effects of curcumin and 5-AZA via JAK2/STAT3 and Nrf2/HO-1 pathways in MCAO mice

We have previously observed that treatment with curcumin and 5-AZA in MCAO mice induces corresponding changes in ADRB2, JAK2/STAT3, and Nrf2/HO-1 pathways. Our earlier in vitro cell experiments demonstrated that ADRB2 can regulate both the JAK2/STAT3 and Nrf2/HO-1 pathways. In this study, we further investigated whether ADRB2 acts as an upstream regulator of JAK2/STAT3 and Nrf2/HO-1 in our in vivo animal model.

**Fig. 9:**
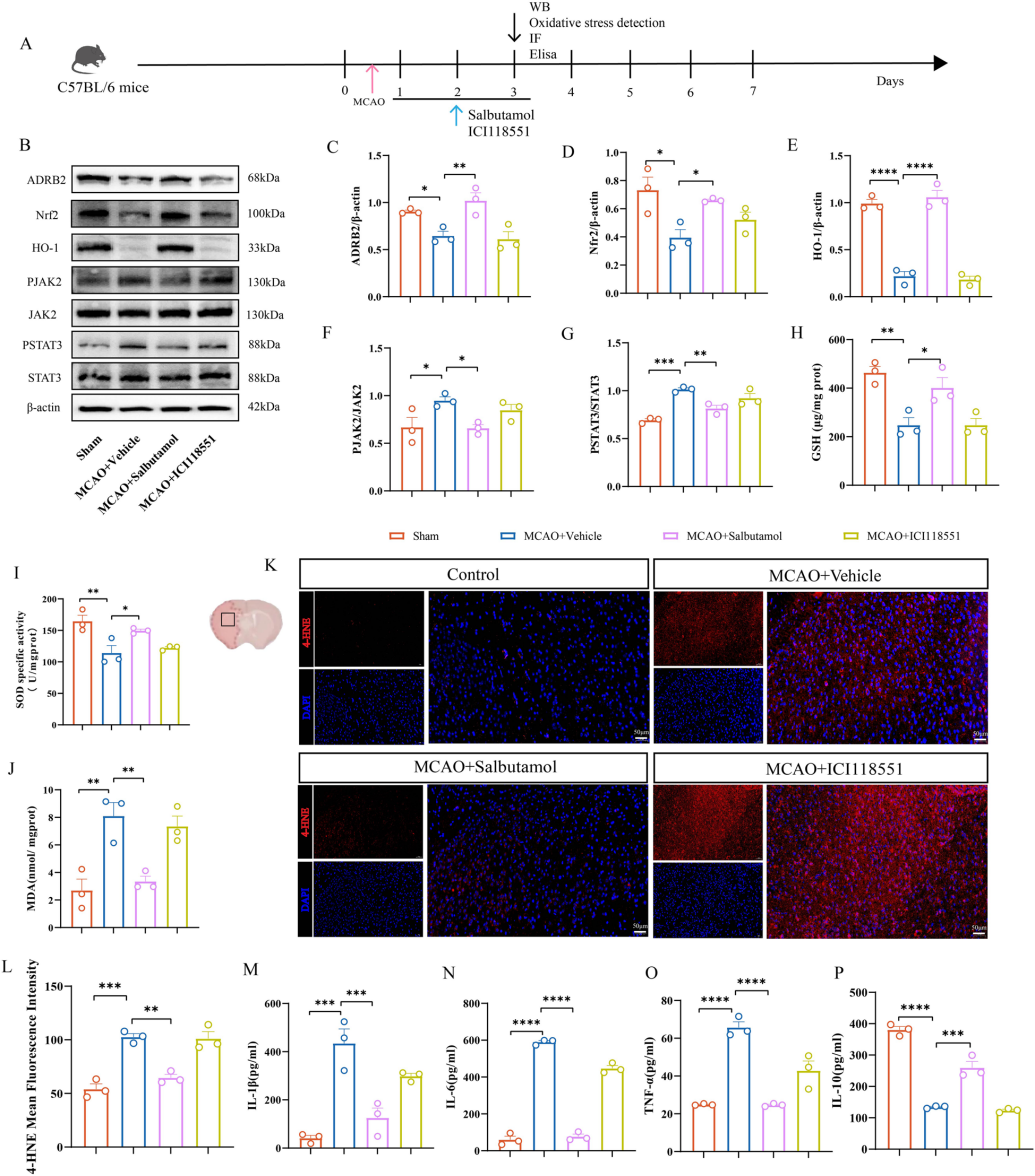
Validation of the correlation between ADRB2 and JAK2/STAT3 and Nrf2/HO-1 signaling in MCAO mice. **A** Flowchart. **B** Representative Western blot bands for ADRB2, p-JAK2, JAK2, p-STAT3, STAT3, Nrf2, and HO-1 in brain tissue lysates from the infarcted area of MCAO mice treated with DMSO, the ADRB2 agonist Salbutamol, or the ADRB2 antagonist ICI118551. The relative levels of each protein were normalized to β-actin from the same sample for Western blot analysis. (C-G) Quantification of ADRB2 (**C**), Nrf2 (**D**), HO-1 (**E**), p-JAK2 and JAK2 (**F**), p-STAT3 and STAT3 (**G**) protein densities. *n* = 3. **H**-**J** Expression levels of oxidative stress markers GSH (**H**), SOD (**I**), and MDA (**J**) in the infarcted area of MCAO mice treated with DMSO, Salbutamol, or ICI118551. *n* = 3. **K** Representative immunofluorescence images of the infarcted area in MCAO mice treated with DMSO, Salbutamol, or ICI118551, with red indicating fluorescence intensity of 4-HNE protein. Scale bar, 50 μm (**L**) Quantification of the average fluorescence intensity of 4-HNE. *n* = 3. **M**-**P** ELISA analysis of inflammatory cytokines IL-1β (**M**), IL-6 (**N**), TNF-α (**O**) and IL-10 (**P**) in brain tissue homogenates from the infarcted area of MCAO mice treated with DMSO, Salbutamol, or ICI118551. *n* = 3. All data are presented as means ± SEM, with statistical analysis performed using one-way ANOVA, and post hoc Dunnett’s test to correct for *P*-values. *P < 0.05, **P < 0.01, ***P < 0.001, ****P < 0.0001

First, after treatment with the ADRB2 agonist salbutamol and the antagonist ICI118551, the expression levels of ADRB2 protein increased and decreased, respectively (Fig. 9B-C). This direct modulation of ADRB2 protein expression by salbutamol and ICI118551 contrasts with the mechanism of curcumin and 5-AZA, as the latter do not influence *Adrb2* methylation directly through receptor binding (Fig. S4C-D). Subsequently, Western blot analysis revealed that in the MCAO + Vehicle group, the expression of Nrf2 and HO-1 decreased. However, after treatment with salbutamol, the expression of both Nrf2 and HO-1 significantly increased (Fig. 9B and D-E). Similarly, salbutamol treatment significantly reduced p-JAK2 in MCAO mice (Fig. 9B and F-G). In addition to protein expression, downstream oxidative stress markers also responded to ADRB2 activation: GSH and SOD levels were elevated (Fig. 9H-I), while MDA levels decreased (Fig. 9J). Immunofluorescence staining further showed that the activation of ADRB2 by salbutamol led to a significant decrease in the fluorescence intensity of 4-HNE (Fig. 9K-L), which confirmed the reduction of oxidative stress. ELISA analysis further proved that after salbutamol treatment, the levels of pro-inflammatory cytokines (IL-1β, IL-6, TNF-α) decreased significantly (Fig. 9M-O), while the levels of anti-inflammatory cytokines (IL-10) increased significantly (Fig. 9P).These results strongly suggest that ADRB2 acts as an upstream factor regulating both the JAK2/STAT3 and Nrf2/HO-1 pathways in vivo, playing a crucial role in modulating neuroinflammation and oxidative stress changes after ischemia-reperfusion.

### Curcumin improves neuroinflammation and oxidative stress by affecting the JAK2/STAT3 axis and Nrf2/HO-1 axis through its action on ADRB2

**Fig. 10:**
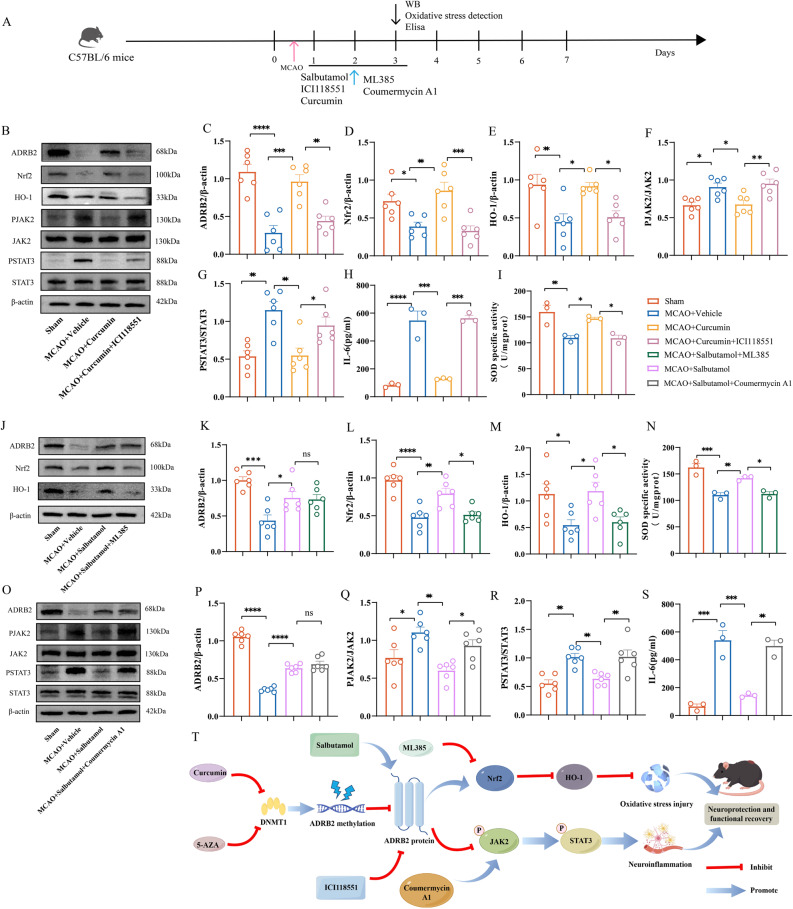
The targets of curcumin are ADRB2, JAK2/STAT3, and Nrf2/HO-1 axis. **A** Flowchart. **B** Representative Western blot bands for ADRB2, p-JAK2, JAK2, p-STAT3, STAT3, Nrf2, and HO-1 in brain tissue lysates from the infarcted area of MCAO mice treated with DMSO, Curcumin, and the ADRB2 antagonist ICI118551. The relative levels of each protein were normalized to β-actin from the same sample for Western blot analysis. **C**-**G** Quantification of ADRB2 (**C**), Nrf2 (**D**), HO-1 (**E**), p-JAK2 and JAK2 (**F**), p-STAT3 and STAT3 (**G**) protein densities. *n* = 6. **H** ELISA analysis of inflammatory cytokines IL-6 in brain tissue homogenates from the infarcted area of MCAO mice treated with DMSO, Curcumin, and the ADRB2 antagonist ICI118551. *n* = 3. **I** Expression levels of oxidative stress markers SOD in the infarcted area of MCAO mice treated with DMSO, Curcumin, and the ADRB2 antagonist ICI118551. *n* = 3. **J** Representative Western blot bands for ADRB2, Nrf2, and HO-1 in brain tissue lysates from the infarcted area of MCAO mice treated with DMSO, Salbutamol, and ML385. The relative levels of each protein were normalized to β-actin from the same sample for Western blot analysis. **K**-**M** Quantification of ADRB2 (**K**), Nrf2 (**L**), HO-1 (**M**) protein densities. *n* = 6. **N** Expression levels of oxidative stress markers SOD in the infarcted area of MCAO mice treated with DMSO, Salbutamol, and ML385. *n* = 3. **O** Representative Western blot bands for ADRB2, p-JAK2, JAK2, p-STAT3 and STAT3 in brain tissue lysates from the infarcted area of MCAO mice treated with DMSO, Salbutamol, and Coumermycin A1. The relative levels of each protein were normalized to β-actin from the same sample for Western blot analysis. **P**-**R** Quantification of ADRB2 (**P**), p-JAK2 and JAK2 (**Q**), p-STAT3 and STAT3 (**R**) protein densities. *n* = 6. **S** ELISA analysis of inflammatory cytokines IL-6 in brain tissue homogenates from the infarcted area of MCAO mice treated with DMSO, Salbutamol, and Coumermycin A1. *n* = 3. **T** Schematic diagram of various drug targets. All data are presented as means ± SEM, with statistical analysis performed using one-way ANOVA, and post hoc Tukey’s test to correct for *P*-values. *P < 0.05, **P < 0.01, ***P < 0.001, ****P < 0.0001

To further demonstrate that the target of curcumin is ADRB2 and that ADRB2 exerts its effects through the JAK2/STAT3 axis and Nrf2/HO-1 axis, we conducted phenotype recovery experiments. After treatment with curcumin, the expression level of ADRB2 protein increased. After administration of ICI118551, the elevated ADRB2 protein was inhibited, while the contents of p-JAK2 and p-STAT3 increased, the expression levels of Nrf2 and HO-1 decreased, the expression of downstream inflammatory factors increased, and the oxidative protective factor SOD decreased (Fig. 10B and I). Subsequently, we administered the Nrf2 specific inhibitor ML385 while stimulating ADRB2, and found that downstream HO-1 and SOD were inhibited after administration of ML385 (Fig. 10J and N). Similarly, while stimulating ADRB2, we administered the JAK2 agonist Coumermycin A1 and found an increase in downstream STAT3 and IL-6 levels (Fig. 10O and S). These results fully demonstrate that the anti-inflammatory and antioxidant effects of curcumin are largely mediated by the ADRB2 protein, which further activates the downstream Nrf2/HO-1 axis and inhibits the JAK2/STAT3 axis. The targets of various drugs are shown in Fig. 10T.

## Discussion

**Fig. 11:**
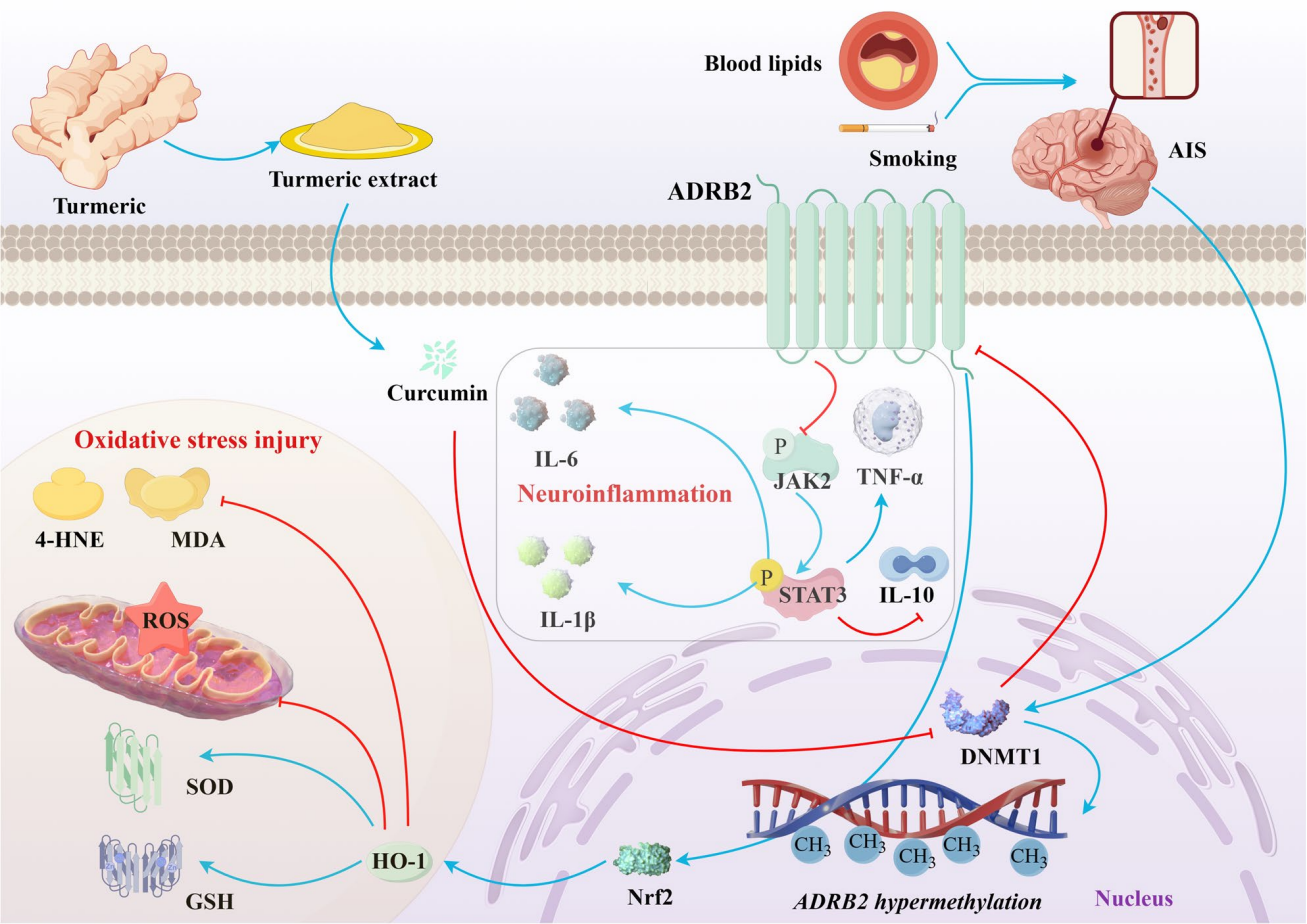
Possible mechanism by which *ADRB2* methylation mediates the progression of AIS. Smoking and dyslipidemia damage intracranial blood vessels, leading to vascular stenosis and promoting the occurrence of AIS. AIS induces the expression of DNMT1, resulting in hypermethylation of the *ADRB2* gene, which leads to decreased protein expression of ADRB2. The inhibitory effect of ADRB2 on JAK2 is weakened, thereby activating STAT3 and promoting the expression of inflammatory cytokines IL-6, IL-1β, and TNF-α. On the other hand, the expression of Nrf2 and HO-1 decreases, while the expression levels of oxidative stress markers 4-HNE, MDA, and ROS increase, and the levels of antioxidant factors SOD and GSH decrease, leading to oxidative stress damage. Curcumin can regulate *ADRB2* promoter methylation, affecting ADRB2 protein levels, and in turn, reduce the expression of IL-1β, IL-6, and TNF-α via the JAK2/STAT3 pathway, while alleviating oxidative stress damage through the Nrf2/HO-1 pathway. The blue arrow represents promotion and the red arrow represents inhibition. *ADRB2* β2-Adrenergic Receptor, *AIS* Acute ischemic stroke, *MDA* Malondialdehyde, *ROS* ‌Reactive Oxygen Species, *SOD* Superoxide Dismutase, *GSH* Glutathione, *HO-1* Hemeoxygenase, *IL-6* Interleukin-6, *IL-1β* Interleukin-1β, *IL-10* Interleukin-10, *TNF-α* Tumor Necrosis Factor-α, *STAT3* Signal transducer and activator of transcription 3, *JAK2* Janus Kinase 2, *DNMT1* DNA methyl transferase 1, *Nrf2* Transcription factor nuclear factor-erythroid 2-related factor 2, *4-HNE* 4-Hydroxynonenal

In this study, we first found that the DNA methylation level of *ADRB2* in AIS patients was significantly higher than that in normal individuals in clinical blood samples, leading to a decrease in ADRB2 protein expression and affecting the function of its encoded β2-adrenergic receptor. At the same time, we found that plasma inflammatory factors were elevated in AIS patients, and the degree of *ADRB2* methylation provided predictive value for the risk of AIS. This suggests that the methylation level of *ADRB2* may serve as a potential biomarker for the diagnosis or prognostic evaluation of acute ischemic stroke. In both in vivo and in vitro experiments, we found that curcumin can target the methylation of *ADRB2* by inhibiting DNMT1 for demethylation, reversing the high methylation state of *ADRB2* in AIS, increasing the expression of ADRB2 protein, activating Nrf2/HO-1 and reversing the inhibition of p-JAK2 and p-STAT3, exerting partial anti-inflammatory and antioxidant activity, thereby reducing the infarct volume of MCAO mice, improving neurological function status, promoting neurological recovery and vascular repair and recanalization (Fig. 11).

DNA methylation is an epigenetic marker involved in regulating genome function and is also the most widely studied epigenetic mechanism [29]. Methylation within gene promoters typically leads to transcriptional silencing, thereby impacting protein function [30]. In ischemic stroke, genes related to neuroprotection, inflammation, and apoptosis may be silenced due to methylation, thereby affecting their function [31, 32]. The exact pathogenesis of AIS is still complex and not fully understood. However, significant advances in epigenetic research have provided new approaches to understand its underlying pathological mechanisms, with DNA methylation being the central focus [33]. Previous studies have shown that DNA methylation plays a key role in the occurrence and outcome of AIS, possibly by promoting cell death and abnormal vascular phenotypes to affect disease progression [34, 35]. As mentioned earlier, previous studies reported a potential association between *ADRB2* and the development of IS [17, 18]. In addition, DNA methylation has also been implicated in the pathogenesis of AIS. Therefore, we focused on *ADRB2* methylation in the context of AIS. In this study, we also found that high methylation poses adverse risks to AIS, especially *ADRB2* methylation. It is worth noting that *ADRB2* is closely related to cells, proliferation, immune regulation, invasion, and angiogenesis, and also mediates inflammation [36]. This is consistent with our findings that low expression of *ADRB2* mediates the occurrence of neuroinflammation after AIS. This is mainly due to the high methylation of *ADRB2* after AIS. Both in vivo and in vitro experiments have found that low expression of *ADRB2* after AIS leads to a decrease in anti-inflammatory and angiogenic abilities. Thus, linking sympathetic nervous system signals with neuroinflammation after cerebral ischemia-reperfusion. In our study, we utilized plasma for semi-quantitative analysis of peripheral ADRB2 levels. While plasma contains soluble receptors such as sIL-1R and sVEGFR [37, 38], which may confound experimental results. However, our target protein ADRB2 is a membrane-bound receptor. Given that soluble plasma receptors generally have minimal interference with membrane proteins, we used transferrin, a stably expressed plasma protein, as an internal reference for semi-quantitative normalization of ADRB2 expression. Interestingly, our study revealed a significant upregulation of IL-10 cytokines in AIS patients. As a well-characterized anti-inflammatory factor, according to the existing literature, IL-10 usually shows decreased expression levels in inflammatory diseases [39]. However, this observation is not absolute, as IL-10 expression may initially increase as part of a compensatory anti-inflammatory response to counter systemic inflammation during the early stages of inflammation. In later stages, however, its anti-inflammatory function becomes impaired, leading to decreased expression [40]. In addition, whether IL-10 has pro-inflammatory or anti-inflammatory effects may depend on specific circumstances, including the cell types involved, the presence of other stimuli or factors, and the overall inflammatory environment [41, 42].

Previous studies have shown that male sex, smoking, alcohol consumption, diabetes, hyperkalemia, and homocysteine are risk factors for cerebral infarction, while ApoA1 is a protective factor. There are gender differences in the methylation of *ABCG1* and *APOE* and the risk of ischemic stroke, with males having a higher risk [43]. Smoking significantly increases the risk of stroke and shows a dose-response relationship, which may be related to abnormal methylation of CpG sites [44–47]. Alcohol is associated with a “J-shaped” or “U-shaped” risk of stroke, and excessive alcohol consumption may increase risk by affecting DNA methylation and oxidative stress [48–50]. Diabetes increases the risk of stroke by 2–4 times, and hyperglycemia and DNA methylation interact [51–53]. Homocysteine aggravates stroke risk by promoting atherosclerosis and neuroinflammation [54]. In addition, decreased levels of ApoA1 are associated with an increased risk of ischemic stroke [55, 56]. The results of our study are basically consistent with previous research, confirming that male gender, smoking, drinking, diabetes, homocysteine are positively correlated with cerebral infarction and negatively correlated with ApoA1. These results suggest that these established risk and protective factors may affect the DNA methylation of the *ADRB2* gene, thereby influencing the susceptibility to ischemic stroke. It is worth noting that our research also found that elevated blood potassium levels may affect *ADRB2* methylation, which may influence cerebral infarction. We hypothesize that this might occur by indirectly regulating the DNA methylation process by disrupting the intracellular/extracellular ionic balance, enzyme activity and signaling pathways. However, given the limited research exploring the relationship between potassium levels and stroke, a larger sample size is needed for further verification. Furthermore, although ApoA1 showed significant differences between the control group and the stroke group and was associated with *ADRB2* methylation, in our cohort, there were no significant differences in TG and ApoE levels between the groups. The levels of TC, LDL cholesterol and ApoB in stroke patients are relatively low, which may be attributed to the continuous use of lipid-lowering drugs in the patient population. Lp(a) showed significant differences between groups, but there was no correlation with *ADRB2* methylation.

Previous studies on central nervous system (CNS) inflammation have focused on the role of microglia [57].In contrast, our study uniquely employed HBMECs as an in vitro model to specifically investigate vascular endothelial inflammatory responses. Existing evidence suggests that vascular endothelium actively releases inflammatory factors during cerebrovascular disease, and these inflammatory responses in turn exacerbate endothelial dysfunction [58]. JAK2/STAT3 signaling pathway is one of the major signaling pathways involved in inflammation, responsible for cytokine production, recruitment and activation of immune cells [59]. After JAK2 activation, STAT3 is phosphorylated, and increased levels of p-JAK2 and p-STAT3 lead to enhanced release of inflammatory factors [60]. Numerous studies have shown that JAK2/STAT3 signaling plays an important role in the inflammatory response caused by neurological diseases, such as cerebral hemorrhage and ischemic stroke [61, 62]. However, the exact relationship between ADRB2 proteins and JAK2 is complicated because they belong to different signaling pathways, GPCR and JAK-STAT, respectively, and the direct interaction has not been fully elucidated. However, there is significant crosstalk between GPCR and JAK-STAT pathways. ADRB2 activation may inhibit JAK2 activity through intermediate molecules. For example, SOCS (suppressor of cytokine signaling) proteins are negative regulators of the JAK-STAT pathway, and ADRB2 may inhibit JAK2 by inducing SOCS expression [63]. Of note, our experimental data strongly demonstrate that both ADRB2 agonist (salbutamol) treatment and ADRB2 overexpression significantly attenuate the p-JAK2 and p-STAT3. These findings strongly suggest that ADRB2 signaling functionally acts upstream of JAK2/STAT3 and that ADRB2 activation may directly inhibit JAK2 phosphorylation to exert a negative regulatory effect. However, further experimental validation is needed to definitively define the precise mechanistic relationships in this crosstalk and the intermediate molecules that may be involved.

Studies have shown that 5-AZA, as a DNA methyltransferase inhibitor, can inhibit inflammation-induced DNA methylation, thereby improving chronic inflammation in tumors [64]. The anti-inflammatory effect of curcumin has been widely studied and has been reported in SAH and stroke patients [24, 28, 65, 66]. Our experiments also confirmed that 5-AZA and curcumin have anti-neuroinflammatory effects after ischemic stroke. However, in our experiment, we further improved the anti-inflammatory mechanism of curcumin by exerting anti-inflammatory activity through epigenetic regulation. It is worth noting that our experiments showed that treatment with either 5-AZA or curcumin led to corresponding changes in the expression level of TET1 protein. TET1 is a DNA demethylase that regulates gene expression [67]. The process involving TET1 and DNMT-mediated methylation represents a dynamic equilibrium. However, in our experiment, both drugs led to a decrease in TET1 expression. Due to the current scarcity of literature on the kinetics of direct linking 5-AZA, curcumin and TET1, we hypothesize that after these drugs inhibit DNMT, cellular mechanisms may down-regulate TET1 expression as a compensatory mechanism to maintain the overall dynamic balance of methylation. However, under these conditions, the main results still favor the demethylation process, which is mainly driven by DNMT inhibition. In our experiment, 5-AZA and curcumin treatments seemed to exhibit considerable therapeutic effects, significantly reducing inflammation and oxidative stress. However, our study mainly focuses on curcumin because 5-AZA is more commonly used as an anti-tumor drug in clinical Settings, and its considerable side effects may have a significant impact on the body. In addition, the methylation regulation of 5-AZA is very clear, while the epigenetic regulation of curcumin remains unclear. In this study, 5-AZA was mainly used to compare the epigenetic regulation of curcumin. Although 5-AZA has shown relevant therapeutic effects, further preclinical studies and clinical trials are needed to evaluate the extent of its benefits for AIS patients. In contrast, curcumin has been reported more frequently for its anti-inflammatory and antioxidant properties. This study explores the anti-inflammatory and antioxidant effects of curcumin in the context of its demethylation mechanism. Therefore, our research focuses more on the role of curcumin. Curcumin may play a role through multiple targets, and changing the epigenetic regulation of *ADRB2* is the key rather than exclusive mechanism in the protection of stroke. However, it is worth noting that the low bioavailability of curcumin limits its wide use and reduces the therapeutic effect [68]. Recent studies have shown that curcumin encapsulated in the lipid core is easy to penetrate the lipid bilayer [69]. Therefore, the purpose of this study is not to claim that curcumin has the conditions for direct clinical application, but to clarify that epigenetic regulation of *ADRB2* can be used as a new target strategy for stroke treatment. This strategy can be extended to other drug designs with better bioavailability, or to optimize the delivery system to improve the bioavailability of curcumin, which is also our key research direction in the future.

Nrf2 is a key transcription factor regulating the expression of antioxidant genes [70]. HO-1, as an important antioxidant enzyme, its expression is regulated by Nrf2 [71, 72]. Studies have shown that ADRB2 may first activate cAMP and then activate the PKA/CREB pathway to affect the expression of Nrf2/HO-1 [73, 74]. Our research provides supportive evidence for this hypothesis, demonstrating that activating ADRB2 or overexpressing it leads to an increase in Nrf2/HO-1 levels, suggesting a potential functional correlation. Research on the direct relationship between 5-AZA and oxidative stress remains limited. Existing evidence indicates that 5-AZA inhibits DNA methyltransferase, reduces DNA methylation levels and reactivates the expression of silenced genes, which in turn can activate the Nrf2 pathway and upregulate the expression of ARE and HO-1 to exert antioxidant effects - a finding confirmed by Chen et al. [75]. Our research found that although 5-AZA treatment could up-regulate Nrf2/HO-1 expression and improve oxidative stress indicators (reduce MDA and ROS, increase SOD and GSH), the difference was not statistically significant, which might be related to drug dose limitation or BBB. In contrast, curcumin demonstrated significant antioxidant effects in both animal and cell models: it significantly increased the expression of Nrf2/HO-1, enhanced the activity of antioxidant factors (SOD, GSH), and reduced oxidative damage products (MDA, ROS). The mechanism may be related to the inhibition of DNMT1 mediated demethylation. The antioxidant stress response of curcumin is consistent with previous studies, but we have further explored new mechanisms to affect the expression of oxidative stress factors [76, 77]. Notably, studies on HO-1 expression changes after cerebral ischemia-reperfusion show inconsistent findings. Qiao and Peng’s research demonstrated reduced HO-1 expression, consistent with our results [78, 79]. Conversely, Sun et al. reported elevated HO-1 levels post-reperfusion [80]. This discrepancy may stem from differences in cell types and HO-1’s dynamic expression patterns. During early reperfusion, HO-1 may temporarily decrease, gradually peak with increased Nrf2 levels, and then slowly decline to exert antioxidant effects [81].

### Limitations

Our study has certain limitations. First, due to the limited sample size in our clinical analysis, we primarily investigated the relationship between *ADRB2* methylation levels, ADRB2 protein expression, and plasma inflammatory factors. We did not measure plasma oxidative stress levels or conduct in-depth research on the specific mechanisms regulating plasma inflammatory factors, which require further verification in subsequent experiments. Meanwhile, due to the inaccessibility of human brain tissue, we are unable to verify the consistency of *ADRB2* methylation between peripheral blood and brain tissue. Although cerebrospinal fluid may more closely reflect the pathophysiological state of brain tissue, obtaining cerebrospinal fluid from AIS patients in clinical practice remains relatively challenging, as they typically do not require this procedure. In addition, we only studied the changes in DNA methylation of peripheral blood leukocytes and did not use cell changes in cerebrospinal fluid to support our viewpoint. In fact, our current data does not clarify the molecular pathways involved, mainly due to the challenge of not being able to obtain normal human tissue samples. In future work, we plan to collect samples of cerebrospinal fluid from patients for further validation. Second, due to technical constraints, we did not perform *Adrb2* gene knockout or overexpression in our in vivo animal experiments to validate the observed phenotypic changes directly. Instead, we confirmed the phenotype and pathway alterations primarily through *ADRB2* overexpression and siRNA knockdown in in vitro cell experiments. The critical in vivo validation of these genetic manipulations remains a necessary step for future studies. Notably, only *ADRB2* overexpression led to statistically significant changes in phenotype and signaling pathways in vitro. Although siRNA-mediated knockdown of *ADRB2* showed an increased trend in markers of inflammation and oxidative stress, these results did not reach statistical significance, although successful *ADRB2* knockdown was confirmed by Western blotting and PCR. We speculate that this may be because after *ADRB2* knockdown, compensatory mechanisms involving other genes or pathways are activated, partially offsetting the loss of its anti-inflammatory and antioxidant effects [82]. Finally, our study did not explore direct mechanistic interactions between *ADRB2* and downstream pathways. For example, we only preliminarily validated functional correlations between *ADRB2* and JAK2 and between *ADRB2* and Nrf2, without confirming whether they physically interact directly or through specific intermediate molecules. Further mechanistic studies, such as co-immunoprecipitation or protein-protein interaction assays, are needed to fully elucidate these detailed molecular relationships.

## Conclusions

In conclusion, our results indicate that DNA methylation levels of the *ADRB2* gene are increased in AIS patients, accompanied by a decrease in ADRB2 protein content. This finding suggests that *ADRB2* gene methylation may serve as a potential biomarker for the diagnosis or prognostic evaluation of AIS. We further demonstrate that the partial anti-inflammatory and antioxidant activities of curcumin can effectively increase the expression of ADRB2 protein in MCAO mice by promoting demethylation. This leads to a reduction in infarct volume and improved neurological recovery. Our mechanistic studies suggest that this neuroprotective mechanism is achieved through the modulation of JAK2/STAT3 and Nrf2/HO-1 pathways. This preclinical study, emphasizing the epigenetic regulation of ADRB2, paves the way for potentially effective therapies for human AIS.

## Supplementary Information

Supplementary Material 1.

## Data Availability

No datasets were generated or analysed during the current study.
