# Supplementary material for: Curcumin reduces neuroinflammation and oxidative stress in a stroke model by epigenetically regulating ADRB2 methylation through JAK2/STAT3 and Nrf2/HO-1 pathways
Source: J Neuroinflammation. 2026 Feb 11;23:109. doi: 10.1186/s12974-026-03729-y (PMC13041036; doi:10.1186/s12974-026-03729-y)
Supplement: Supplementary file 1 — Supplementary Material 1. [file 12974_2026_3729_MOESM1_ESM.docx]

**Supplementary Methods**

**Clinical Participant Recruitment**

The patients with AIS in this study were all hospitalized in the Department of Neurology of the First Affiliated Hospital of Ningbo University from January 2024 to August 2024, totaling 90 cases. The control group consisted of 90 hospitalized patients who were excluded from acute cerebrovascular disease, including hemifacial spasm, trigeminal neuralgia, Parkinson's disease, etc. After strict gender and age matching. Inclusion criteria for this study: Age≥18, regardless of gender; Diagnosed as AIS according to the Chinese Guidelines for the Diagnosis and Treatment of Acute Ischemic Stroke 2023;Arrive or be admitted within 6 hours after the onset of symptoms; First clinical attack of cerebral infarction without serious sequelae; Modified Rankin Scale (mRS) score≤1 before stroke onset; During screening, the National Institutes of Health Stroke Scale (NIHSS) score ranged from 8 to 20(inclusive), and the NIHSS item 1a (level of consciousness) score was≤1;The subject or their legal guardian has signed an informed consent form. Exclusion criteria for this study: Acute phase of cerebral hemorrhage and other hemorrhagic diseases; Pre stroke disabled patients; Indications for anticoagulant therapy before diagnosis, such as atrial fibrillation, and the use of medication; In addition to using drugs for hypertension and diabetes, other diseases require long-term use of drugs; Diagnosed with severe mental illness: moderate or severe depression before stroke, functional impairment, and suicide risk; Important medical history within the past 5 years: severe cardiovascular disease, severe infectious disease, severe liver or kidney failure, newly diagnosed or actively treated cancer. The initial diagnosis was made by a junior doctor and later confirmed by a senior doctor. All patients signed informed consent forms and obtained approval from the Ethics Review Committee of the First Affiliated Hospital of Ningbo University.

**Clinical Data Collection**

Comprehensive clinical information was collected for each enrolled participant through detailed medical history interviews. This included demographic data (gender, age, body mass index [BMI]), admission blood pressure, history of hypertension and diabetes, smoking and alcohol consumption status, and relevant biochemical indicators (blood lipids, glucose levels) and hematological parameters (e.g., white blood cell count).

**Blood Sample Collection and Biochemical Analysis**

All patients were taken to the hospital by ambulance at the time of onset, and two 5mL blood samples were collected from all patients within 2 hours of admission. One 5mL tube was used for immediate biochemical analysis. Levels of total cholesterol (TC), triglyceride (TG), low-density lipoprotein (LDL), high-density lipoprotein (HDL), apolipoprotein A1 (ApoA1), apolipoprotein B (ApoB), apolipoprotein E (ApoE), and lipoprotein (a) (Lp(a)) were measured using an automatic biochemical analyzer (Olympus AU2700, Tokyo, Japan) at the hospital's biochemical laboratory. Another 3mL blood sample was centrifuged at 3000 rpm for 10 minutes at 4°C to collect plasma, which was then stored at -80°C for subsequent PCR, ELISA, and other experiments, including the detection of plasma inflammatory factors. The remaining 2ml of whole blood was centrifuged and leukocytes were obtained to extract genomic DNA for methylation analysis.

**Pyrosequencing** **for DNA Methylation Analysis**

For quantitative DNA methylation analysis, genomic DNA was extracted from peripheral blood leukocytes using the Qiagen genomic DNA extraction kit (Cat. No. BJD4068, Hilden, Germany). Extracted DNA was then subjected to bisulfite conversion. Specific primer sequences for *ADRB2* pyrosequencing were designed using PyroMark Assay Design software and are provided in Supplementary Table 2. This software was also used to select 10 CpG sites within the target region for quantitative analysis of methylation levels. These 10 CpG sites were selected for detecting *ADRB2* methylation in human peripheral blood and cell samples. For animal tissues and peripheral blood, five additional CpG sites were specifically designed to assess *Adrb2* methylation.

**Detection of Plasma Cytokines**

Plasma levels of cytokines (IL-2, IL-4, IL-6, TNF-α, IFN-γ, IL-10, and IL-17) in AIS patients and control subjects were measured using the Cytometric Bead Array (CBA) method. The CBA procedure was performed following the manufacturer’s instructions using a human Th1/Th2/Th17 cytokine kit (flow cytometric Luminex assay) (Cat. No. P010701001, Saiji Biotechnology, Nanchang, China). The specific method of CBA is as follows: We completed it based on the manufacturer's reagent kit. Firstly, vortex the microspheres for 20 seconds, mix thoroughly, centrifuge at 200×g for 5 minutes, discard the supernatant, and add an equal volume of microsphere buffer. Incubate at room temperature in a dark environment for 15 minutes, vortex and mix for 20-30 seconds, and add 25μL microspheres to each experimental tube. After adding 25μL of test sample and 25μL of fluorescent detection reagent, incubate in the dark for 2.5 hours. After incubation, add 1 mL of PBS buffer to each well, centrifuge at 200×g for 5 minutes, discard the supernatant, and add 100μL of PBS buffer to each well before testing on the machine. Cytokine levels were subsequently analyzed using a flow cytometer (BD Biosciences, NJ, USA).

**Cell Culture**

Human brain microvascular endothelial cells (HBMECs) were obtained from Zhong Qiao Xin Zhou Biotechnology Co., Ltd. (Shanghai, China; Catalog No. ZQ0961). Cells were cultured in Endothelial Cell Medium (ECM) (Cat. No. 36608, ScienCell Research Laboratories, San Diego, CA, USA), supplemented with 1% penicillin/streptomycin, 5% fetal bovine serum (FBS), and appropriate amounts of endothelial cell growth supplement (ECGS). Cells were maintained in a humidified incubator at 37°C with 5% CO₂. Upon reaching 80-90% confluence, typically after 3-4 days of culture, cells were passaged. The culture medium was refreshed every 2-3 days to ensure adequate nutrient supply.

**Establishment of OGD/R Model and Drug Treatment**

To establish the in vitro oxygen-glucose deprivation/reoxygenation (OGD/R) cell model, HBMECs were washed three times with glucose-free, serum-free DMEM medium before the old culture medium was discarded. Cells were then cultured in glucose-deprived DMEM medium and placed in a hypoxic incubator containing a gas mixture of 1% O₂, 5% CO₂, and 94% N₂ to achieve oxygen deprivation. An optimal OGD duration was determined by treating cells at 37°C for 2, 4, and 6 hours. Following OGD treatment, the medium was replaced with normal ECM containing 5% FBS, and cells were subjected to reoxygenation for 24 hours under normoxic conditions (5% CO₂ and 95% air) at 37°C. Control cells were cultured in complete medium under normoxic conditions throughout the experiment. Based on previous literature, 5μM 5-AZA (Cat. No. HY-A0004, MedChemExpress, New Jersey, USA) was selected for subsequent in vitro studies (21). For curcumin treatment, 7.5μM curcumin (Cat. No. HY-N0005, MedChemExpress, New Jersey, USA) was chosen based on subsequent cell viability assays combined with its efficacy in inhibiting DNMT1. Additionally, 100nM salbutamol (Cat. No. HY-B1037, MedChemExpress, New Jersey, USA) (22) and 25μM ICI118551 (Cat. No. B1004, APExBIO, Houston, Texas, USA) (23) were used as per literature. After OGD/R, cells were co-incubated with 5-AZA, curcumin, salbutamol, and ICI118551 for 24 hours prior to further analysis. The experimental groups in the cell-based studies were designed as follows:(1) Control group: HBMECs without any treatment; (2) OGD/R group: HBMECs subjected to oxygen-glucose deprivation (OGD) for 4 hours followed by reoxygenation (R) for 20 hours; (3) OGD/R + 5-AZA group: HBMECs treated with 5-AZA for 24 hours after OGD (4 h) and reoxygenation (20 h); (4) OGD/R + curcumin group: HBMECs treated with curcumin for 24 hours after OGD (4 h) and reoxygenation (20 h); (5) OGD/R + *ADRB2* Plasmid group: HBMECs transfected with the *ADRB2* plasmid before OGD (4 h) and reoxygenation (20 h); (6) OGD/R + *ADRB2* siRNA group: HBMECs transfected with *ADRB2* siRNA before OGD (4 h) and reoxygenation (20 h); (7) OGD/R + Salbutamol group: HBMECs treated with salbutamol for 24 hours after OGD (4 h) and reoxygenation (20 h); (8) OGD/R + ICI 118551 group: HBMECs treated with ICI 118551 for 24 hours after OGD (4 h) and reoxygenation (20 h). Throughout the experiment, salbutamol served as an agonist of the ADRB2 protein, ICI 118551 as a highly selective antagonist of ADRB2, and 5-AZA as a well-established demethylating agent to investigate whether curcumin exerts demethylation effects in comparison.

**Animal Handling and Ethics**

Male C57BL/6 mice, weighing 24-26 g, were acquired from Zhejiang Vital River Laboratory Animal Technology Co. Ltd. All animal care and experimental procedures strictly adhered to the guidelines established by the Institutional Animal Care and Use Committee of Ningbo University, which also provided ethical approval for this study. To minimize sex-related variables, only male C57BL/6 mice were used. Animals were housed under controlled environmental conditions (22-24°C, 40-60% humidity) with a 12-hour light/dark cycle and provided ad libitum access to food and water.

**Establishment of MCAO Model and Drug Treatment**

The middle cerebral artery occlusion (MCAO) model was established in mice as previously described. Mice were anesthetized via intraperitoneal injection of 1.25% Tribromoethanol. Upon loss of corneal reflex and resistance, they were secured on a surgical board in a supine position. The neck area was shaved and disinfected. A 2-3 cm midline incision was made to expose the right common carotid artery (CCA). Through careful blunt dissection between the sternocleidomastoid and sternohyoid muscles, the arterial sheath was exposed, taking care to avoid damaging the trachea. The right CCA was isolated from the vagus nerve, and both the internal carotid artery (ICA) and external carotid artery (ECA) were subsequently dissected. The proximal ends of the CCA and ECA were ligated. The distal CCA was temporarily occluded using a weighted suture, and a loose knot was prepared proximal to this occlusion site. A small incision was then made between this loose knot and the proximal CCA ligation point for the introduction of a filament. The filament was gently advanced to the occlusion site, the loose knot was tightened, and the temporary distal CCA occlusion was released to allow further advancement. Filament insertion was stopped when the marker reached approximately 2 mm past the CCA bifurcation or upon encountering slight resistance. After confirming hemostasis, the skin was sutured with approximately 2 cm of the filament left externalized. Reperfusion was established by withdrawing the filament after 1 hour of occlusion. Control mice, designated as the sham group, underwent identical anesthesia and surgical procedures but without MCAO. All mice were randomly allocated into the following groups: Sham, MCAO + Vehicle, MCAO + Curcumin, MCAO + 5-AZA, MCAO + Salbutamol, and MCAO + ICI118551. Drug administration commenced immediately at 1-hour post-reperfusion and continued for 3 consecutive days via intraperitoneal injection according to established protocols: 5-AZA group: 0.12 mg/kg (21), Curcumin group: 150 mg/kg (24), Salbutamol group: 15 mg/kg (22), ICI118551 group: 1mg/kg (25). According to the literature, the dosage of ML385 (Cat. No. B8300, APExBIO, Houston, Texas, USA) is 30 mg/kg (26), and the dosage of Coumermycin A1(Cat. No. HY-N7452, MedChemExpress, New Jersey, USA) is 100μg/kg (27). Administer once every 24 hours. Drug therapy should be administered within 72 hours after treatment (acute phase), and brain tissue and peripheral blood should be obtained.

**Cerebral Perfusion Imaging**

Cerebral blood perfusion in mice was monitored using a laser speckle contrast imaging system (Simopto, Wuhan, China). After anesthesia, mice were fixed on a bench, and their heads were depilated. The scalp was incised to expose the skull. The laser speckle imager was positioned approximately 200-300 mm from the tested mice. The sampling frequency was set to 5 images/s, with an effective sampling frequency of 1 image/s. During imaging, efforts were made to keep mice still to avoid limb twitching artifacts. Blood perfusion images were collected from each MCAO mouse at three key time points: immediately after filament insertion (occlusion confirmation), immediately after filament withdrawal (reperfusion confirmation), and on the third day post-MCAO. These images were used to evaluate the success of MCAO modeling and the therapeutic effects of drug treatments. Mice exhibiting a reduction in cerebral blood flow (CBF) by ≥30% compared to baseline following filament insertion were considered successful MCAO models and were included in subsequent experiments (28). CBF data were quantified in perfusion units (PU), reflecting relative changes in tissue perfusion.

**Infarct Volume Measurement**

Infarct volume was assessed at the acute phase of cerebral infarction (day 3) using 2,3,5-triphenyltetrazolium chloride (TTC) (Cat. HY-B1102, Solarbio, Beijing, China) staining. Briefly, mice were anesthetized with 1.25% Tribromoethanol and euthanized by decapitation. Brains were rapidly extracted on ice and coronally sectioned into 2 mm-thick slices. These brain slices were then immersed in a 2% TTC solution and incubated at 37°C for 20 minutes, followed by overnight fixation with 4% paraformaldehyde (PFA) at 4°C. Ischemic areas appeared white, clearly delineated from the red-stained normal brain tissue. Infarct volume was quantitatively analyzed using ImageJ software.

**Evaluation of Blood-Brain Barrier Permeability**

Blood-brain barrier (BBB) permeability was evaluated on day 3 after MCAO using Evans Blue (EB) extravasation. Mice were intraperitoneally injected with a 2% EB solution (Cat. No. HY-B1102, MedChemExpress, New Jersey, USA) at a dose of 4 mL/kg and allowed to circulate for 60 minutes. Following this, mice were anesthetized, and transcranial perfusion with phosphate-buffered saline (PBS) was performed to thoroughly remove intravascular dye. Brain tissues were then collected. Macroscopic images of EB extravasation in whole brains were captured first. Subsequently, brains were coronally sectioned into 1 mm-thick slices for photographic documentation of EB leakage patterns. The infarcted brain tissues were carefully dissected and weighed. EB dye was then extracted from the tissue using formamide, and its concentration was quantified by measuring absorbance at 620 nm against a standard curve of known EB concentrations.

**Assessment of Neurological Deficits**

Neurological function in mice was comprehensively evaluated using the modified neurological severity score (mNSS), angle test, rotarod test, and sticky paper removal test. All tests were performed one day before MCAO and then on days 1, 3, 5, and 7 post-MCAO. In all experiments, each animal was independently assessed three times by two researchers blinded to the experimental conditions. To minimize potential confounding effects of fatigue on experimental outcomes, all animals underwent the corner test in the morning of the testing day, followed by the adhesive removal test and mNSS assessment (low-physical-demand task). The rotarod test was then conducted in the afternoon of the same day. The mNSS score was determined based on a comprehensive assessment involving the tail-lifting test, walking test, sensory test, balance beam test, and observations for loss of reflexes and abnormal movements. Scores from each individual test were summed, with a higher total score indicating more severe neurological functional impairment. Angle Test: Mice were placed between two plates forming an included angle. A test was considered complete when the mouse fully lifted its hind legs along the angle and then turned. Each mouse underwent 10 trials, and the number of turns to the left and right was recorded. A significant increase in turns towards the uninjured side was expected in MCAO mice due to hemiplegia. Rotarod Test: Mice were trained for 3 days prior to MCAO. The rotarod apparatus was set to a linear accelerating speed mode, with an initial speed of 10 rpm and a final speed of 30 rpm. Mice were acclimatized with a 10rpm pre-run for 2 cycles before the test commenced. The time each mouse remained on the rotating rod until falling was recorded. Sticky Paper Removal Test: Sticky paper was applied to the paws of both the left and right forelimbs of the mice. The time taken for the mouse to discover and subsequently remove the sticky paper from each paw was observed and recorded.

**Live/Dead Cell Staining**

To assess cell viability after OGD/R, the Calcein-AM/Propidium Iodide (PI) Cell Viability and Cytotoxicity Assay Kit (Cat. C2015M, Beyotime Biotechnology, Shanghai, China) was used. One day before the experiment, cells were trypsinized, counted, and seeded into 24-well plates. Following OGD/R treatment, the reagents provided in the kit were added, and cells were incubated at 37°C for 30 minutes in the dark. Cell viability and cytotoxicity were then evaluated under a fluorescence microscope. Perform cell counting analysis using ImageJ software.

**Cell Viability Test**

To evaluate the effects of different concentrations of 5-AZA or curcumin on cell viability, the Cell Counting Kit-8 (CCK-8) assay (Cat. RM02823, ABclonal, Wuhan, China) was performed. One day prior to the experiment, cells were trypsinized, counted using an automated cell counter, and seeded into 96-well plates. After 24 hours of 5-AZA or curcumin treatment, 10μL of CCK-8 reagent was added to each well, followed by incubation for 4 hours. Absorbance at 450 nm was measured using a microplate reader.

**Cell Transfection**

*ADRB2* siRNA Knockdown: To establish the *ADRB2* small interfering RNA (siRNA) knockdown system, *ADRB2*-targeted siRNA and non-targeting empty siRNA (control) were synthesized by GenePharma. Transfection was performed using Lipofectamine 3000 (Lip3000, Cat. CN2541162, Thermo Fisher Scientific, MA, USA) as the transfection vector. siRNA (at a final concentration of 50nM) and Lip3000 were diluted in Opti-MEM medium. Cells were transfected in Opti-MEM medium for 6 hours, after which the medium was replaced with complete culture medium for further incubation.

*ADRB2* Plasmid Overexpression: To establish the *ADRB2* plasmid overexpression system, an *ADRB2* expression vector was synthesized by GenePharma. Plasmid DNA (at a concentration of 500 ng/μL) was diluted with p3000 and Lipofectamine 3000 in Opti-MEM medium. The subsequent transfection procedure is the same as the siRNA transfection procedure.

**Enzyme-Linked Immunosorbent Assay (ELISA)**

The collected cell culture supernatants and animal brain tissue homogenates were processed according to the manufacturer's instructions. Subsequently, the expression levels of IL-6, IL-10, IL-1β, and TNF-α were measured using species-specific ELISA kits. Human ELISA Kits (for cell culture supernatants): Human IL-6 ELISA Kit (Cat. RK00004, ABclonal, Wuhan, China), Human IL-1β ELISA Kit (Cat. RK00001, ABclonal, Wuhan, China), Human TNF-α ELISA Kit (Cat. RK00030, ABclonal, Wuhan, China), Human IL-10 ELISA Kit (Cat. RK00012, ABclonal, Wuhan, China). Mouse ELISA Kits (for animal brain tissue homogenates): Mouse IL-6 ELISA Kit (Cat. RM17709, ABclonal, Wuhan, China), Mouse IL-1β ELISA Kit (Cat. RM17859, ABclonal, Wuhan, China), Mouse TNF-α ELISA Kit (Cat. RK00027, ABclonal, Wuhan, China), Mouse IL-10 ELISA Kit (Cat. RK00016, ABclonal, Wuhan, China). The absorbance at 450 nm was measured using a microplate reader.

**Western Blotting**

After discarding the culture medium, cell samples were washed three times with cold phosphate-buffered saline (PBS). For animal brain tissue, 0.1 g of brain tissue was weighed and homogenized in RIPA lysis buffer (Cat. R0010, Solarbio, Beijing, China) supplemented with protease and phosphatase inhibitors. Following complete lysis, protein concentrations were determined using the BCA method (Cat. P0012, Beyotime Biotechnology, Shanghai, China). Protein samples were then denatured by boiling at 100°C for 10 minutes. Equal amounts of protein were loaded onto SDS-PAGE gels for electrophoresis. Separated proteins were then transferred to PVDF membranes. Membranes were blocked with 5% skim milk at room temperature for 2 hours, followed by incubation with primary antibodies at 4°C for 12-18 hours. The primary antibodies and their dilutions were: Rabbit anti-ADRB2 antibody (1:2000, ABclonal, Cat. A2048), rabbit anti-p-JAK2 antibody (1:3000, ABcam Cat. ab32101), rabbit anti-JAK2 antibody (1:3000, Abcam, Cat. ab108596), rabbit anti-p-STAT3 antibody (1:3000, Abcam, Cat. ab76315), rabbit anti-STAT3 antibody (1:3000, Abcam, Cat. ab68153), rabbit anti-TNF-α antibody (1:1000, Affinity, Cat.AF7014), rabbit anti-IL-6 antibody (1:1000, Affinity, Cat.DF6087), rabbit anti-IL-1β antibody (1:1000, Affinity, AF5103), rabbit anti-DNMT1 antibody (1:2000, proteintech, Cat.24206-1-AP), rabbit anti-TET1 antibody (1:5000, ABclonal, Cat. A1506), rabbit anti-Nrf2 antibody (1:3000, ABclonal, Cat.A21176), rabbit anti- HO-1 antibody (1:2000, ABclonal, Cat. A1346), mouse anti-Transferrin antibody (1:5000, Affinity, Cat.BF0720), rabbit anti-actin antibody (1:20000, Affinity, Cat.AF7018), mouse anti-actin antibody (1:20000, Affinity, Cat.T0022). Following primary antibody incubation, membranes were washed and then incubated with the appropriate HRP-conjugated secondary antibodies (1:5000 dilution) at room temperature for 1 hour. Protein bands were visualized using a chemiluminescence imager, and semi-quantitative analysis was performed using ImageJ software.

**Real-Time Quantitative Polymerase Chain Reaction (RT-qPCR)**

Total RNA was extracted from samples using the RNA rapid extraction kit (Cat. RN001, ESscience Biotech, Shanghai, China). RNA was then reverse-transcribed into cDNA using ABScript III RT Master Mix (Cat. RK20429, ABclonal, Wuhan, China). Finally, RT-qPCR was performed using PerfectStart® Green qPCR SuperMix (Cat. AQ601, TransGen Biotech, Beijing, China) on a LightCycler 480 system from Roche (Mannheim, Germany). PCR primer sequences are provided in Supplementary Table 2. The 2-^ΔΔ^ CT method was used to calculate the relative expression between the given sample and the reference sample.

**Immunofluorescence Staining**

For cell immunofluorescence, cells were seeded onto cell climbing pieces in perforated plates until they reached approximately 80% confluence. For brain tissue, frozen sections were prepared. Both cell and tissue samples were fixed with 4% paraformaldehyde (Cat. P1110, Solarbio, Beijing, China) for 20 minutes. After thorough washing, samples were permeabilized with 0.5% Triton X-100 (Cat. T8200, Solarbio, Beijing, China) for 30 minutes. Non-specific binding was blocked with immunofluorescence blocking solution (Cat. P0206, Beyotime Biotechnology, Shanghai, China) at room temperature for 30 minutes. Primary antibodies were added and incubated at 4°C for 12-18 hours: Rabbit anti-ADRB2 antibody (1:200, ABclonal, Cat. A2048)，Goat anti-CD31 antibody(1:200, R&D Systems, Cat.AF3628), Rabbit anti-4HNE antibody(1:200,ABclonal, Cat.A26085). Following primary antibody incubation, samples were washed and then incubated with the appropriate fluorescently conjugated secondary antibodies (1:500 dilution) at room temperature for 1 hour. Each climbing slice or frozen section was then stained with DAPI (Cat. S2110, Solarbio, Beijing, China) for 20 minutes to counterstain nuclei. Finally, coverslips were mounted using neutral resin. Fluorescence images were acquired using a fluorescence microscope, and image analysis was performed using ImageJ software.

**Detection of Oxidative Stress Markers**

For cell samples, a defined number of cells were collected and homogenized in PBS. For tissue samples, mouse brain tissues were harvested 72 hours after MCAO and homogenized in cold PBS buffer. Following homogenization, samples were centrifuged, and the supernatant was collected. The levels of lipid peroxidation (Malondialdehyde, MDA), reduced glutathione (GSH), and Superoxide Dismutase (SOD) activity were determined using commercially available kits according to the manufacturer's instructions: Lipid Peroxidation Malondialdehyde (MDA) Determination Kit (Cat. E-BC-K025-ME, Elabscience, Wuhan, China). Reduced Glutathione (GSH) Colorimetric Test Kit (Cat. E-BC-K030-M, Elabscience, Wuhan, China). Superoxide Dismutase (SOD) Activity Determination Kit (Cat. E-BC-K020-M, Elabscience, Wuhan, China). Quantification of these markers was performed using a Microplate Reader. Cellular reactive oxygen species (ROS) content was detected using the CheKine™ Reactive Oxygen Species (ROS) Content Detection Kit (Fluorescence Method) (Cat. KTB1911, Abbkine, Wuhan, China). Specifically, cells were incubated with 10μM fluorescent probe DCFH-DA, which enters cells and is oxidized by intracellular ROS to produce fluorescent DCF. The fluorescence signal intensity of DCF in living cells was observed using a fluorescence microscope. Image analysis was performed with ImageJ to quantify the level of reactive oxygen species based on fluorescence signal intensity.

**Statistical Analysis**

All data were analyzed using GraphPad Prism statistical analysis software (Version 8.3.0, CA, USA). When comparing the two groups of data, the normality test was carried out first. The two groups of normal distribution data were compared by using the two tailed unpaired Student's t test, and the non normal distribution data were tested by Mann - Whitney U rank sum test. When analyzing the data between groups, the homogeneity test of variance is carried out first. If the variance is homogeneous, the one-way analysis of variance is used for the comparison between groups. Dunnett's multiple comparisons test is used to correct P-values. If the variance is not homogeneous, the Kruskal Wallis h analysis method is used. Pearson correlation was used for correlation analysis. Receiver operating characteristic (ROC) curve was used to analyze the predictive value of methylation for cerebral infarction. All data were measured with mean ± standard error (SEM), P<0.05 indicates that the difference is statistically significant.

Supplementary Table 1. Summary of animal numbers and mortality.

| **Groups** | **Mortality** | **Excluded (CBF≤30%)** |
| --- | --- | --- |
| **Part 1: Effects of Curcumin and 5-AZA on Short term Research Results** | | |
| **neuroethology** | | |
| MCAO + Vehicle (n=12) | 0% (0/14) | 2 |
| MCAO+5-AZA (n=12) | 14.3% (2/14) | 0 |
| MCAO + Curcumin (n=12) | 12.5% (2/16) | 2 |
| **Cerebral blood perfusion imaging and TTC** | | |
| MCAO + Vehicle (n=6) | 25% (2/8) | 0 |
| MCAO +5-AZA (n=6) | 12.5% (1/8) | 1 |
| MCAO + Curcumin (n=6) | 14.3% (1/7) | 0 |
| Part 2: Study on the mechanism of reducing neuroinflammation and oxidative stress by inhibiting DNMT1 and reducing ADRB2 methylation through curcumin and 5-AZA after AIS | | |
| **WB** | | |
| Sham (n=6) * | 0% (0/6) | 0 |
| MCAO + Vehicle (n=6) * | 0% (0/6) | 0 |
| MCAO + 5-AZA (n=6) | 0% (0/9) | 3 |
| MCAO + Curcumin (n=6) | 14.3% (1/7) | 0 |
| IF |  |  |
| Sham (n=3) # | 0% (0/3) | 0 |
| MCAO + Vehicle (n=3) # | 20% (1/5) | 1 |
| MCAO+5-AZA (n=3) | 20% (1/5) | 1 |
| MCAO + Curcumin (n=3) | 25% (1/4) | 0 |
| **BBB** | | |
| Sham (n=3) | 0% (0/3) | 0 |
| MCAO + Vehicle (n=3) | 0% (0/3) | 0 |
| MCAO+5-AZA (n=3) | 20% (1/5) | 1 |
| MCAO + Curcumin (n=3) | 25% (1/4) | 0 |
| **Oxidative stress factor detection（GSH，SOD，MDA）** | | |
| Sham (n=3) | 0% (0/3) | 0 |
| MCAO + Vehicle (n=3) | 25% (1/4) | 0 |
| MCAO +5-AZA (n=3) | 0% (0/4) | 1 |
| MCAO + Curcumin (n=3) | 0% (0/3) | 0 |
| **Methylation sequencing of MCAO mice (brain tissue, peripheral blood)** | | |
| Sham (n=12) | 0% (0/12) | 0 |
| MCAO + Vehicle (n=12) | 7.14% (1/14) | 1 |
| MCAO+5-AZA (n=12) | 14.3% (2/14) | 0 |
| MCAO + Curcumin (n=12) | 7.69% (1/13) | 0 |
| MCAO + Salbutamol (n=6) | 0% (0/6) | 0 |
| MCAO + ICI118551 (n=6) | 0% (0/6) | 0 |
| **Part 3: Improvement of neuroinflammation and oxidative stress is achieved through JAK2/STAT3 and Nrf2/HO-1, respectively** | | |
| **WB** | | |
| Sham (n=3) * | 0% (0/3) | 0 |
| MCAO + Vehicle (n=3) * | 0% (0/3) | 0 |
| MCAO + Salbutamol (n=3) | 25% (1/4) | 0 |
| MCAO + ICI118551 (n=3) | 25% (1/4) | 0 |
| IF |  |  |
| Sham (n=3) # | 0% (0/3) | 0 |
| MCAO + Vehicle (n=3) # | 20% (1/5) | 1 |
| MCAO + Salbutamol (n=3) | 20% (1/5) | 1 |
| MCAO+ ICI118551 (n=3) | 25% (1/4) | 0 |
| **ELISA** | | |
| Sham (n=3) | 0% (0/3) | 0 |
| MCAO + Vehicle (n=3) | 0% (0/4) | 1 |
| MCAO + Salbutamol (n=3) | 0% (0/3) | 0 |
| MCAO+ ICI118551 (n=3) | 0% (0/3) | 0 |
| **Oxidative stress factor detection（GSH，SOD，MDA）** | | |
| Sham (n=3) | 0% (0/3) | 0 |
| MCAO + Vehicle (n=3) | 0% (0/3) | 0 |
| MCAO+ Salbutamol (n=3) | 20% (1/5) | 1 |
| MCAO+ ICI118551 (n=3) | 0% (0/3) | 0 |
| **Part 4: Rescue experiment** | | |
| **WB** | | |
| Sham (n=6) | 0% (0/6) | 0 |
| MCAO + Vehicle (n=6) | 0% (0/7) | 1 |
| MCAO + Curcumin (n=6) | 14.3% (1/7) | 0 |
| MCAO + Curcumin + ICI118551 (n=6) | 0% (0/6) | 0 |
| MCAO + Salbutamol (n=6) | 14.3% (1/7) | 0 |
| MCAO + Salbutamol + ML385 (n=6) | 0% (0/7) | 1 |
| MCAO + Salbutamol + Coumermycin A1 (n=6) | 0% (0/6) | 0 |
| **ELISA (IL-6)** | | |
| Sham (n=3) | 0% (0/3) | 0 |
| MCAO + Vehicle (n=3) | 20% (1/5) | 1 |
| MCAO + Curcumin (n=3) | 0% (0/3) | 0 |
| MCAO + Curcumin + ICI118551 (n=3) | 25% (1/4) | 0 |
| MCAO + Salbutamol (n=3) | 0% (0/4) | 1 |
| MCAO + Salbutamol + Coumermycin A1 (n=3) | 0% (0/3) | 0 |
| **Oxidative stress factor detection（SOD）** | | |
| Sham (n=3) | 0% (0/3) | 0 |
| MCAO + Vehicle (n=3) | 0% (0/3) | 0 |
| MCAO + Curcumin (n=3) | 0% (0/3) | 0 |
| MCAO + Curcumin + ICI118551 (n=3) | 25% (1/4) | 0 |
| MCAO + Salbutamol (n=3) | 0% (0/4) | 1 |
| MCAO + Salbutamol + ML385 (n=3) | 25% (1/4) | 0 |
| **Total** |  |  |
| Sham (n=45) | 0.0% (0/45) | 0 |
| MCAO (n=243) | 10.2% (30/294) | 21 |

* Shared groups in WB. # Shared groups in IF.

Supplementary Table 2. Primer sequence.

| Primer | Sequence |
| --- | --- |
| **Pyrosequencing** |  |
| *ADRB2* Human Forward Primer | GAGGGAAAGGGGAGGAGT |
| *ADRB2* Human Reverse Primer | ACCTACCAATTCCAACCC |
| *ADRB2* Human Sequencing Primer | GAAAGGGGAGGAGTG |
| *Adrb2* Mouse Forward Primer | GTTATGTGTATTAGATTTTAGGGGAATT |
| *Adrb2* Mouse Reverse Primer | ACAACCCTAAATTTCTCTCTCTAA |
| *Adrb2* Mouse Sequencing Primer | ATTAGATTTTAGGGGAATTG |
| **RT-qPCR** |  |
| *ADRB2* Forward Primer | TTGCCTCTTCCATCGTGTCC |
| *ADRB2* Reverse Primer | CCACCTGGCTAAGGTTCTGG |
| *IL-10* Forward Primer | TTGCTGGAGGACTTTAAGGGTT |
| *IL-10* Reverse Primer | TCACATGCGCCTTGATGTCT |
| *1L-6* Forward Primer | CAGCGATGATGCACTGTCA GA |
| *1L-6* Reverse Primer | TCAATAGGCAAATTTCCTGGTTAT ATC |
| *IL-1β* Forward Primer | GGACAGGATATGGAGCAACAAGTGG |
| *IL-1β* Reverse Primer | TCATCTTTCAACACGCAGGACAGG |
| *TNF -α* Forward Primer | GAGGCCAAGCCCTGGTATG |
| *TNF -α* Reverse Primer | CGGGCCGATTGATCTCAGC |
| *ACTB* Forward Primer | CATGTACGTTGCTATCCAGGC |
| *ACTB* Reverse Primer | CTCCTTAATGTCACGCACGAT |


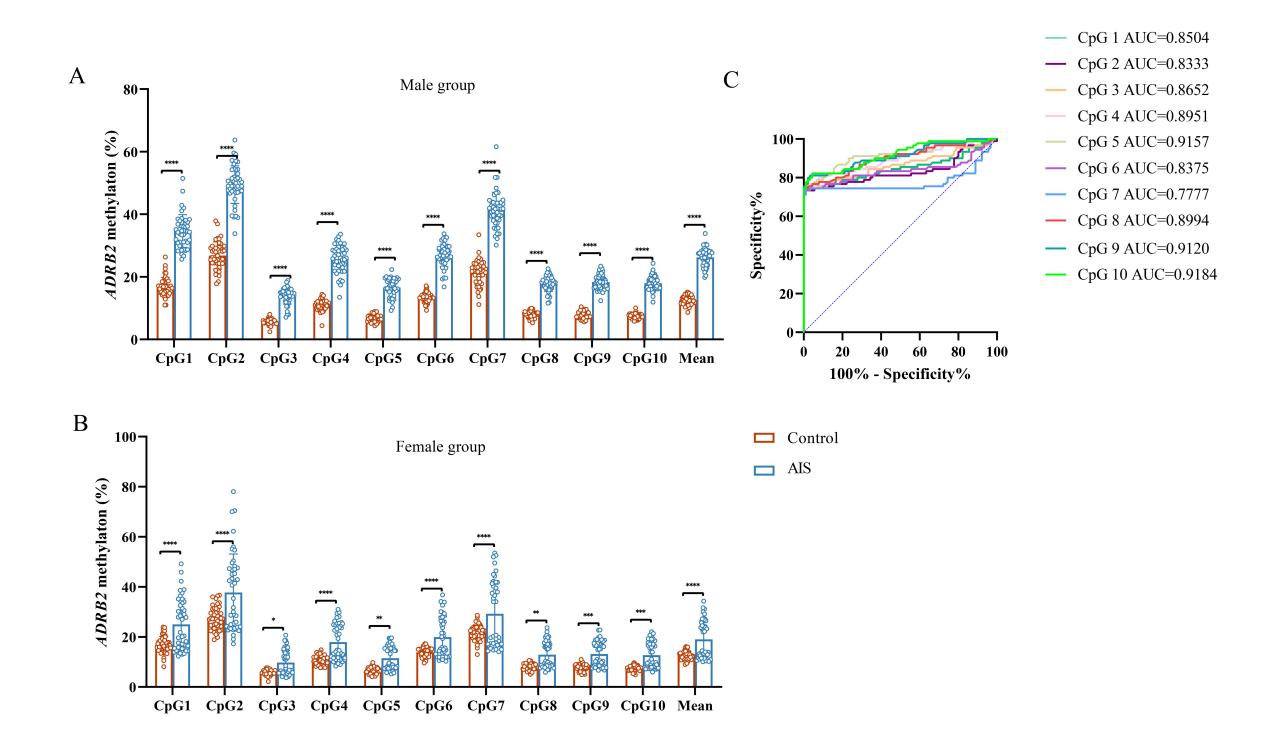


Supplementary Figure 1: Analysis of the relationship between gender and *ADRB2* CpG10 site methylation. (A) Methylation differences of the *ADRB2* CpG10 site between the control and AIS groups in males. (B) Methylation differences of the *ADRB2* CpG10 site between the control and AIS groups in females. (C) Receiver operating characteristic (ROC) curve of *ADRB2* CpG1-10 methylation. Data are presented as means ± SEM and analyzed using the Mann-Whitney test. *P < 0.05, **P < 0.01, ***P < 0.001, ****P < 0.0001, n = 45.





Supplementary Figure 2: Correlation analysis between *ADRB2* gene methylation and clinical data. (A) Scatter plot of the correlation between K^+^ and *ADRB2* CpG7 site methylation. (B) Scatter plot of the correlation between K^+^ and *ADRB2* CpG10 site methylation. (C) Scatter plot of the correlation between HCY and *ADRB2* CpG2 site methylation. (D) Scatter plot of the correlation between HCY and *ADRB2* CpG7 site methylation. (E) Scatter plot of the correlation between HCY and *ADRB2* CpG6 site methylation. (F) Scatter plot of the correlation between ApoA1 and *ADRB2* CpG3 site methylation. (G) Scatter plot of the correlation between ApoA1 and *ADRB2* CpG4 site methylation. (H) Scatter plot of the correlation between ApoA1 and *ADRB2* CpG5 site methylation. (I) Scatter plot of the correlation between ApoA1 and *ADRB2* CpG7 site methylation. (J) Scatter plot of the correlation between ApoA1 and *ADRB2* CpG8 site methylation. (K) Scatter plot of the correlation between ApoA1 and *ADRB2* CpG9 site methylation. (L) Scatter plot of the correlation between ApoA1 and *ADRB2* CpG10 site methylation. (M) Scatter plot of the correlation between ApoA1 and the average methylation of all *ADRB2* CpG sites. All data were analyzed using Pearson correlation analysis, with R representing the correlation coefficient. n = 90.


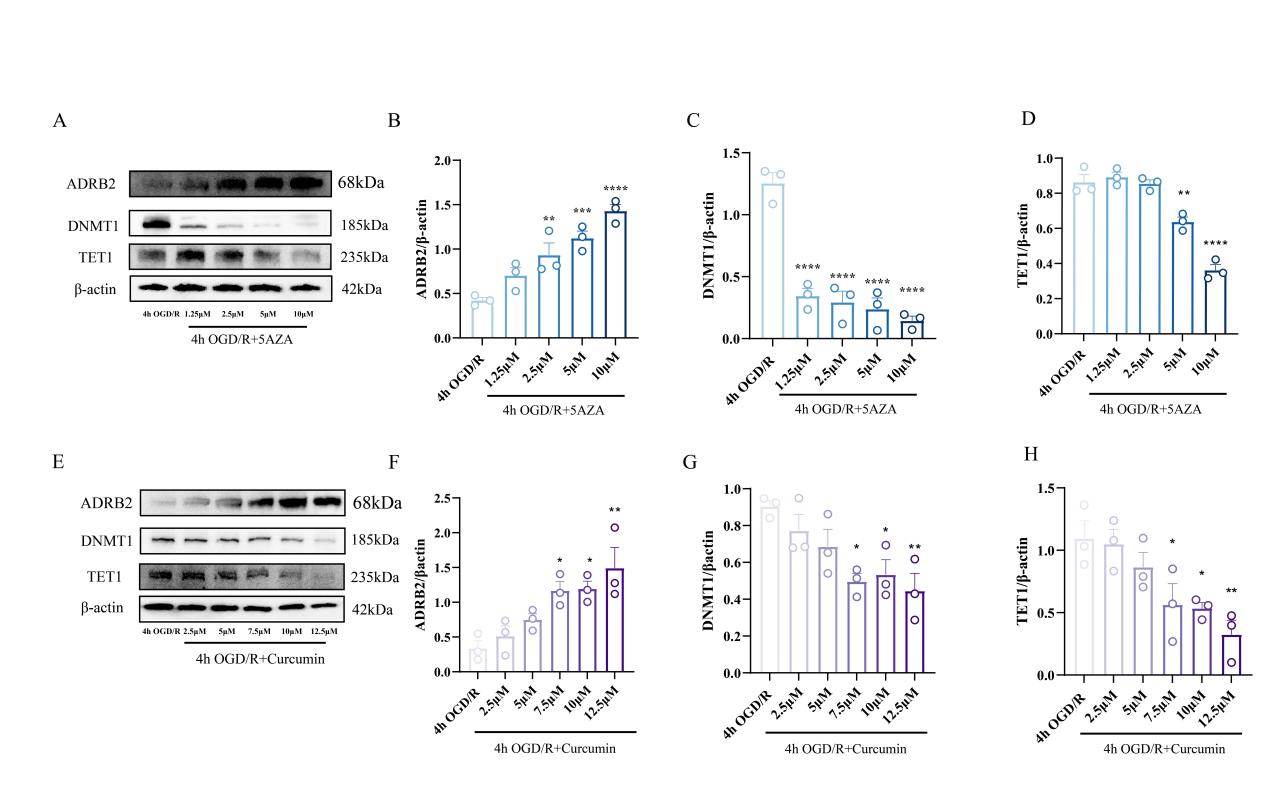


Supplementary Figure 3: Effects of curcumin and 5-AZA concentration gradients on ADRB2, DNMT1, and TET1 protein expression. (A) Representative western blot bands for ADRB2, DNMT1, and TET1 in HBMECs lysates treated with different concentrations of 5-AZA. The relative levels of proteins were normalized to β-actin from the same sample for western blot analysis. (B-D) Quantification of the protein density of ADRB2 (B), DNMT1 (C), and TET1 (D) following treatment with different concentrations of 5-AZA. n = 3. (E) Representative western blot bands for ADRB2, DNMT1, and TET1 in HBMECs lysates treated with different concentrations of curcumin. The relative levels of proteins were normalized to β-actin from the same sample for western blot analysis. (F-H) Quantification of the protein density of ADRB2 (F), DNMT1 (G), and TET1 (H) following treatment with different concentrations of curcumin. n = 3. All data were analyzed using one-way ANOVA, and post hoc Dunnett’s test to correct for P-values. All data presented as means ± SEM. *P < 0.05, **P < 0.01, ***P < 0.001, ****P < 0.0001, n = 3.


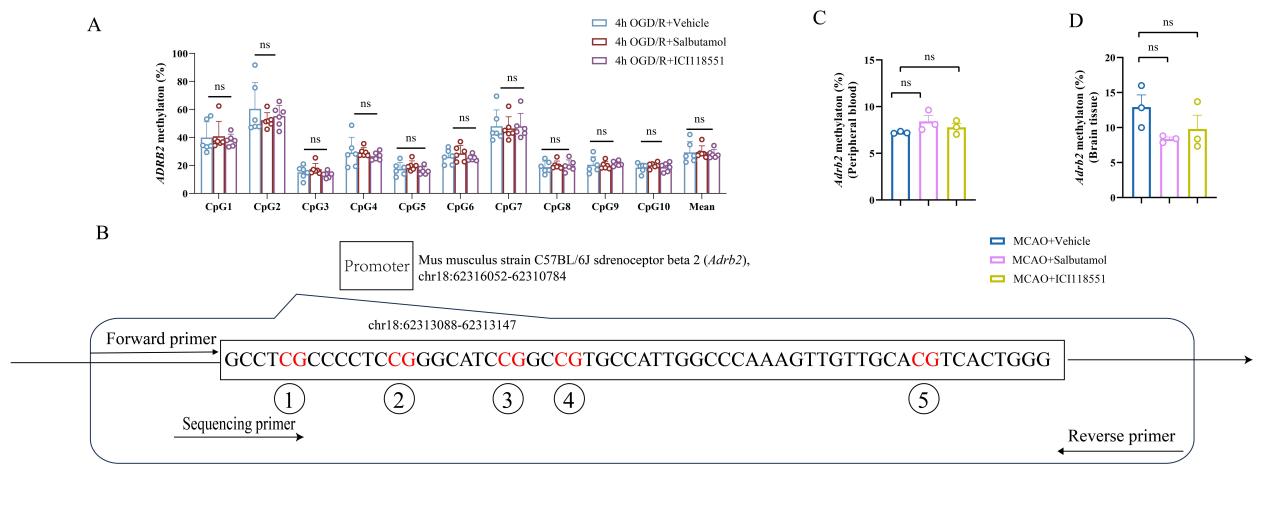


Supplementary Figure 4: ADRB2 agonist Salbutamol and ADRB2 antagonist ICI118551 do not affect *ADRB2* methylation. (A) The methylation levels of *ADRB2* CpG sites 1-10 in HBMECs subjected to OGD/R induction, treated with solvent DMSO, ADRB2 agonist Salbutamol, and ADRB2 antagonist ICI118551, were assessed by pyrosequencing. n=6. (B) Schematic representation of the *Adrb2* target sequence within the promoter region of the mouse genome, showing the distribution of the five CpG sites. (C) Methylation levels of the *Adrb2* CpG island in the infarct area of MCAO mice peripheral blood treated with DMSO, ADRB2 agonist Salbutamol, and ADRB2 antagonist ICI118551 were evaluated by pyrosequencing. (D) Methylation levels of the *Adrb2* CpG island in the infarct area of MCAO mice brain tissue treated with DMSO, ADRB2 agonist Salbutamol, and ADRB2 antagonist ICI118551 were evaluated by pyrosequencing. n=3. All data are presented as means ± SEM and analyzed using one-way ANOVA, and post hoc Dunnett’s test to correct for P-values. *P < 0.05, **P < 0.01, ***P < 0.001, ****P < 0.0001. The data in Figures C and D is shared with the data in Figures 8P-Q of the main text
